# Targeted gene knockin in zebrafish using the 28S rDNA-specific non-LTR-retrotransposon R2Ol

**DOI:** 10.1186/s13100-019-0167-2

**Published:** 2019-05-22

**Authors:** Azusa Kuroki-Kami, Narisu Nichuguti, Haruka Yatabe, Sayaka Mizuno, Shoji Kawamura, Haruhiko Fujiwara

**Affiliations:** Department of Integrated Biosciences, Graduate School of Frontier Sciences, The University of Tokyo, Bioscience Bldg., Kashiwanoha 5-1-5, Kashiwa, Chiba, 277-8562 Japan

**Keywords:** Non-LTR retrotransposon, Long interspersed element (LINE), 28S rDNA specific element R2, Sequence-specific retrotransposition, Targeted gene knockin, Transgenic zebrafish

## Abstract

**Background:**

Although most of long interspersed elements (LINEs), one class of non-LTR-retrotransposons, are integrated into the host genome randomely, some elements are retrotransposed into the specific sequences of the genomic regions, such as rRNA gene (rDNA) clusters, telomeric repeats and other repetitive sequenes. Most of the sequence-specific LINEs have been reported mainly among invertebrate species and shown to retrotranspose into the specific sequences in vivo and in vitro systems. Recenlty, 28S rDNA-specific LINE R2 elements are shown to be distributed among widespread vertebrate species, but the sequence-specific retrotransposition of R2 has never been demonstrated in vertebrates.

**Results:**

Here we cloned a full length unit of R2 from medaka fish *Oryzias latipes*, named R2Ol, and engineered it to a targeted gene integration tool in zebrafish. By injecting R2Ol-encoding mRNA into zebrafish embryos, R2Ol retrotransposed precisely into the target site at high efficiency (98%) and was transmitted to the next generation at high frequency (50%). We also generated transgenic zebrafish carrying the enhanced green fluorescent protein (EGFP) reporter gene in 28S rDNA target by the R2Ol retrotransposition system.

**Conclusions:**

Sequence-specific LINE retrotransposes into the precise sequence using target primed reverse transcription (TPRT), possibly providing an alternative and effective targeted gene knockin method in vertebrates.

**Electronic supplementary material:**

The online version of this article (10.1186/s13100-019-0167-2) contains supplementary material, which is available to authorized users.

## Background

Precisely targeted gene integration technologies that can enable transgene expression while avoiding unpredictable phenotypes caused by random integration are desirable. In terms of the safety aspect, sequence-specificity is the most important subject associated with transgene integration at targeted harmless genomic sites for therapeutic purposes in human cells [1]. Clustered regularly interspaced short palindromic repeats / CRISPR associated proteins (CRISPR/Cas9) system is the most powerful and prevalent genetic engineering tool system which induces double-strand breaks (DSBs) at the target site, stimulating two cellular DNA repair pathways: non-homologous end-joining (NHEJ) and homology directed repair (HDR). The error-prone NHEJ process generates small insertions or deletions at the DSB site, causing knockout of the target sequence. Alternatively, HDR results in gene knockin at the target site in the presence of donor DNA. The HDR efficiency is relatively low compared with NHEJ [2, 3], and designing the donor DNA is time-consuming. Some strategies to increase HDR efficiency, such as utilization of nickase or dead Cas9 (dCas9) [4, 5], inhibition of NHEJ [6, 7], usage of asymmetric donor DNA [8], and cell cycle synchronization [9], have been employed. However, the molecular mechanism of donor DNA template-dependent HDR requires further elucidation [3], and off-target concerns [10] as well as cancer risk [11, 12] should be monitored. Thus, another system independent of HDR might provide an alternative solution for efficiently targeted gene knockin.

Long interspersed elements (LINEs), one class of non-LTR-retrotransposon, encode reverse transcriptase (RT) and endonuclease (EN) domains that nick one strand of DNA at the target site to create a 3′-hydroxyl end, which is used as a primer for the reverse transcription of LINE mRNA into the DNA target [13]. This unique process, called target-primed reverse transcription (TPRT), is peculiar to LINEs. Although many LINEs, such as human L1, retrotranspose almost randomly into the genome, there are various sequence-specific LINEs that integrate with specific sequences within a target site, such as rDNA, telomeric repeats, microsatellites, and others [14–16]. In vivo sequence-specific retrotransposition systems for some sequence-specific elements, such as telomere-specific SART1 and TRAS1 [17], 28S rDNA-specific R1Bm [18], and 18S rDNA-specific R7Ag [19], have been established in insect cells using a baculovirus vector. The most studied sequence-specific element R2Bm of the silkworm, also exhibited the efficient sequence-specific retrotransposition activity both in vitro [13] and in vivo [20], contributing to understanding the general mechanism of LINE retrotransposition. The target specificity of these elements is quite rigid, making them suitable for targeted gene integration tools in widespread species [14], although no system has been reported in vertebrates. Most sequence-specific LINEs have been detected only in arthropod and invertebrate species. However, we have recently found that 28S rDNA-specific R2 is widely distributed among vertebrate species, but not mammals [16, 21]. It is noteworthy that all R2 elements found in various species are inserted into the same sequence within 28S rDNA, an approximately 200 bp region surrounded by strongly conserved sequences across a wide variety of eukaryote [14] (Additional file 1: Fig. S1), suggesting the high sequence specificity of this element.

## Results

### R2Ol retrotransposes accurately and efficiently in zebrafish

**Fig. 1:**
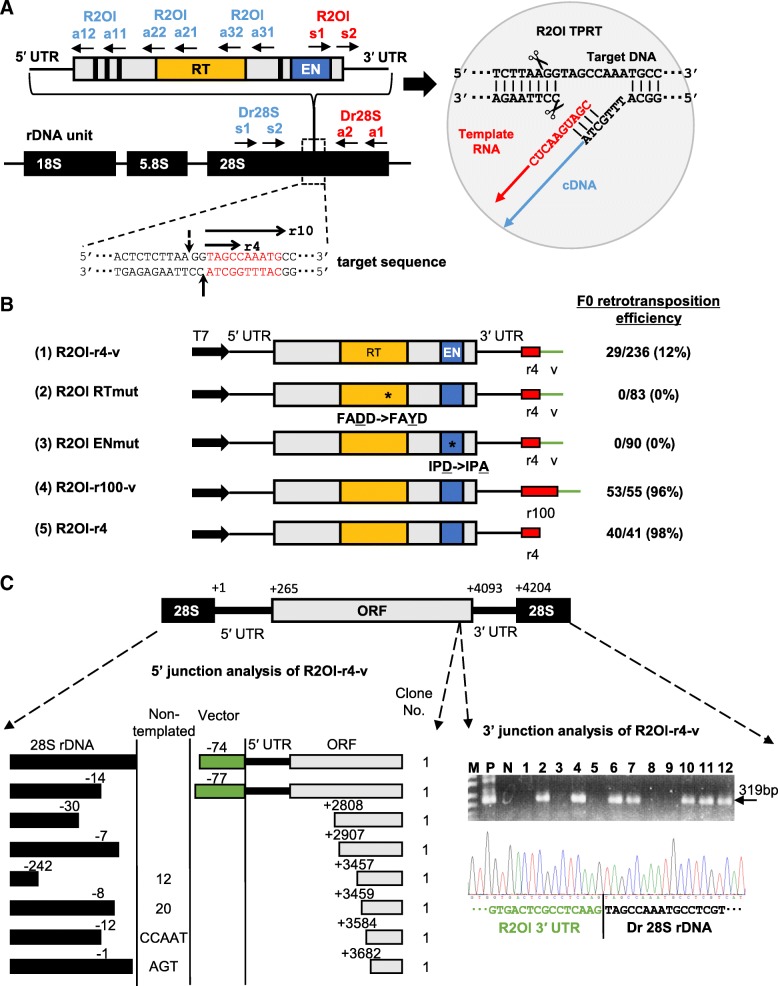
R2Ol retrotransposition assay in zebrafish. **a** Left panel: R2Ol ORF is indicated by a gray closed rectangle and the 5′ and 3′ untranslated region (UTR) by horizontal lines. The approximate positions of the reverse transcriptase (RT) and endonuclease (EN) are indicated. Vertical lines represent the cysteine-histidine motifs. Horizontal arrows indicate the primers used to detect retrotransposition events. The rDNA unit is indicated by a black closed rectangle. The double-strand sequences of the precise insertion sites are indicated. The bottom and putative top strand cleavage sites are indicated by solid and dashed line, respectively. In the downstream 28S sequence of R2Ol, 1 to 4 (TAGC) and 1 to 10 (TAGCCAAATG) nucleotides are denoted as r4 and r10, respectively, and shown by red letters and horizontal arrows. Right panel: A schematic diagram of the R2Ol ribonucleoprotein (RNP) complex and target primed reverse transcription (TPRT) mechanism. R2Ol mRNA (red) anneals with the target DNA (28S rDNA) at the 3′ junction to initiate cDNA (blue) synthesis. **b** Schematic diagram of the R2Ol mRNA constructs used in the retrotransposition assay. T7, T7 promoter; red box, 28S rDNA; green line, vector (v) sequence; (2) R2Ol RTmut, RT mutant of FADD- > FAYD and (3) R2Ol ENmut, EN mutant of IPD- > IPA (mutation indicated by the dashed line). F0 retrotransposition efficiency for each construct are indicated on the right (see also Table 1). **c** 3′ and 5′ junction PCR assay of inserted R2Ol for retrotransposition detection. Right panel: 3′ junction PCR results of R2Ol-r4-v. P, positive-control PCR with medaka fish genomic DNA; N, negative-control PCR with uninjected zebrafish genomic DNA; 1–12, injected samples. 3′ junction sequence of the R2Ol retrotransposition events showed in the bottom. Green indicates R2Ol and black indicates 28S rDNA sequences. Left Panel: 5′ junction analysis of inserted R2Ol-r4-v. Green boxes indicate vector sequences, respectively. Non-templated indicates extra sequences at the junction derived from neither 28S rDNA nor R2Ol. Truncated or deleted sites are indicated by nucleotide numbers. The clone number of each insertion type is indicated in the far-right column

**Fig. 2:**
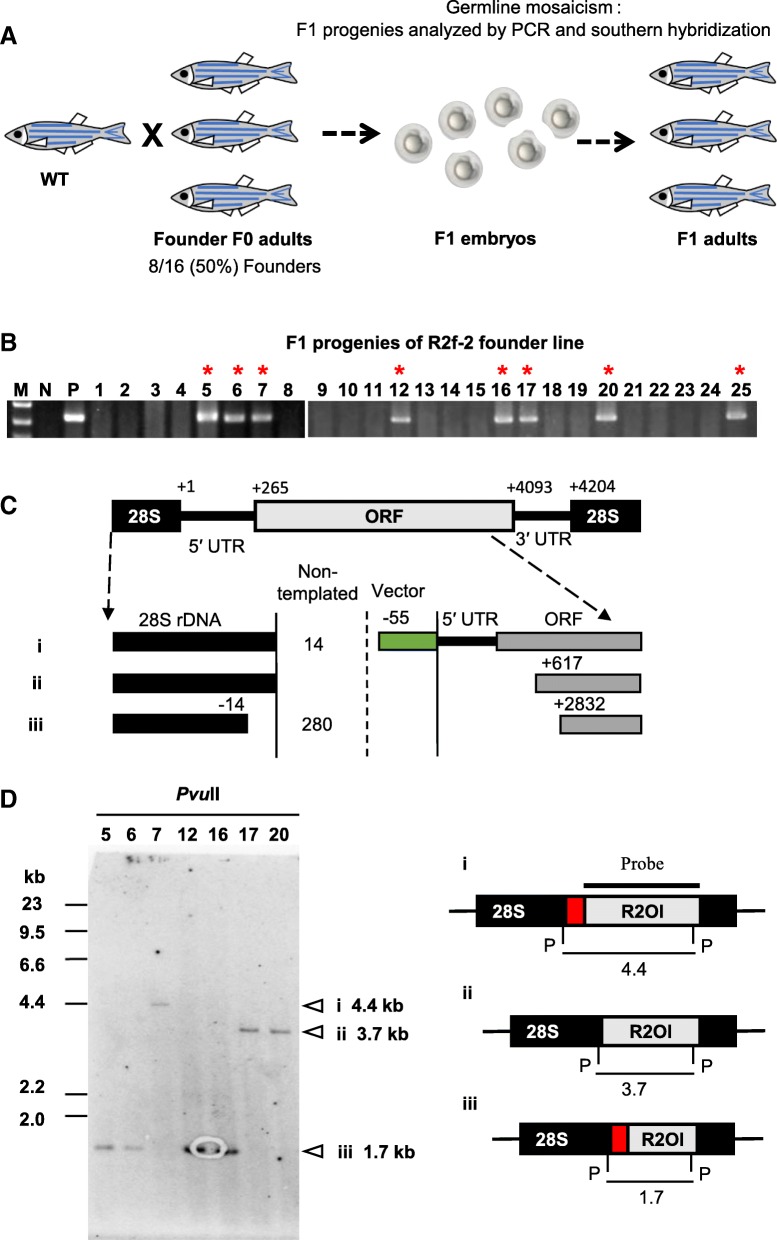
Germline transmission of R2Ol in zebrafish. **a** A Founder F0 fish was crossed with WT to obtain F1 progenies. F1 embryos or adults were used for the germline mosaicism analysis. **b** PCR screening of 3′ junction of R2Ol-positive fish (Red asterisks) using genomic DNA from F1 caudal fins. **c** 5′ junction sequence of inserted R2Ol. Black box, 28S rDNA sequence; Gray box, R2Ol sequence; Vector, vector sequence; Non-templated, non-template sequence. Truncated or deleted sites are indicated by nucleotide numbers. **d** Left panel: Southern blot analysis of PCR-positive F1 fish. Right panel: Schematic diagram of three detected R2Ol insertions. P, PvuII site; thick line, probe; thin lines and numbers, signal lengths. Red boxes indicate vector or non-templated sequence

First, we cloned a full-length unit of R2Ol from medaka genomic DNA by polymerase chain reaction (PCR) (see Methods). Among the nine genomic clones obtained, we selected clone F (Accession number: LC349444), which contained minimal amino acid changes in comparison with the consensus sequence, to use for further analyses (Additional file 1: Figure S2 A, B). The R2Ol unit comprises a 5′ untranslated region (UTR; 265 bp), a single open reading frame (ORF; 3831 bp) encoding EN and RT domains, and a 3′ UTR (108 bp) (Fig. 1a, Additional file 1: Figure S2C). A capped mRNA was transcribed from a full-length R2Ol construct in vitro and injected into one-cell zebrafish embryos to verify the retrotransposition activity (Additional file 1: Figure S3-i). To detect the sequence-specific retrotransposition, we extracted genomic DNA from the injected embryos and performed nested PCR to amplify the 3′ junction between R2Ol and 28S rDNA utilizing specific primer sets (Fig. 1a, red primers). After sequencing the PCR fragment, we judged the sequence-specific integration and calculated the retrotransposition efficiency. This direct mRNA injection method was advantageous in that we did not need to consider the plasmid transcriptional efficiency by comparing the retrotransposition activity of each construct. We expected that a 4-bp 28S rDNA flanking sequence (r4) at the 3′-side of R2Ol would increase the annealing efficiency between the template RNA and target DNA [18, 20] (Fig. 1a, right panel). Thus, the R2Ol construct initially used for retrotransposition analyses contained the r4 sequence followed by the 302 bp vector sequence (v) just downstream of the 3′ UTR, which included an SV40 poly-A signal sequence (Fig. 1b (1) R2Ol-r4-v). Using this construct, we detected a 319-bp PCR band representing the precise retrotransposition in 29 individuals among 236 injected embryos (12%) (12 representatives of the PCR analyses are shown in (Fig. 1c, Right panel). Sequencing the PCR band revealed that the medaka R2Ol was inserted into the exact target sequence of the heterologous zebrafish 28S rDNA (Fig. 1c, Right panel). In contrast, two constructs mutated at the essential amino acid sites for EN and RT domains, completely abolishing the retrotransposition activities (Fig. 1b, (2) R2Ol RTmut and (3) R2Ol ENmut), suggesting that R2Ol moves by TPRT and not by recombination events. The above results confirmed that the medaka fish R2Ol exhibited the precise and efficient retrotransposition activity into the 28S rDNA target sequence in the heterologous zebrafish host. We further analyzed the 5′-junctions between the retrotransposed R2Ol and the 28S rDNA target using four ORF primers sets (Fig. 1a, blue primers). Of the eight insertions tested, six represented truncated R2Ol, terminating reverse transcription at various sites within the ORF (Fig. 1c, Left panel). This type of 5′-truncation is often observed among LINEs. Notably, the remaining two represented a full-length insertion (> 4.2 kb) of R2Ol, suggesting that longer genes could be knocked-in by this system. To evaluate the retrotransposition efficiency of R2Ol, we elongated the 28S rDNA sequence flanking the 3′ UTR of R2Ol from 4 bp to 100 bp which was expected to anneal more strongly with the target DNA. Using R2Ol-r100-v construct, the efficiency increased to 96% compared with 12% in the R2Ol-r4-v construct (Fig. 1b (4)). We also deleted the vector sequence from the construct (R2Ol-r4), which drastically increased of the retrotransposition efficiency to 98% (Fig. 1b (5)). These results indicated that annealing of the extreme 3′-end of read-through R2Ol mRNA with the target 28S rDNA enhances the retrotransposition activity, and the extra vector sequence in the 3′-end region might rather disturb the initial TPRT step.

### R2Ol is transmitted to next generation of zebrafish

To determine whether the retrotransposed R2Ol-r100-v could be transmitted to germline cells, we raised R2Ol-injected embryos to adulthood and crossed the adult fish with an uninjected wild type (WT) fish to yield F1 embryos (Fig. 1b, (4) R2Ol-r100-v, Fig. 2a, Additional file 1: Figure S3-ii). Genomic DNA was extracted from more than 100 pooled F1 embryos, and a 3′ junction PCR analysis was conducted to identify the founder fish. Founders were identified 50% in F0 fish, indicating that R2Ol was efficiently transmitted to germline cells (Table 1). Next, to determine the germline mosaicism of individual founders, we analyzed the F1 progeny of six founders. In the progenies of the R2f-2 line, 8 of 25 adult fins (Fig. 2b) and 4 of 30 embryos (data not shown) showed positive PCR bands representing the R2Ol insertion, indicating a total germline mosaicism rate of 22% (Table 1). We further analyzed the 5′ junction sequences of the R2Ol insertion and found several different genomic copies caused by 5′-truncation in each progeny line (Table 1). In the R2f-2 line, we detected three types of R2Ol insertion by sequencing: Pattern-i comprised a full-length R2Ol with an upstream vector sequence insertion (Fig. 2c, Additional file 3: Table S2). Pattern-ii and -iii displayed the 5′-truncation at different sites within the R2Ol ORF (Fig. 2c, Additional file 3: Table S2). Southern blot hybridization using DNA from F1 fishes in the R2f-2 line confirmed the above results and, more importantly, did not detect off-target gene knockin in other genomic regions other than rDNA (Fig. 2d). We analyzed two other founders via Southern blot hybridization and detected two and one R2Ol patterns in R2f-9 and R2f-10, respectively (Additional file 1: Figure S4, Table 1 R2Ol-r100-v). All detected R2Ol insertions were located in 28S rDNA, suggesting no off-target integration. In summary, the germline mosaicism rate of R2Ol was calculated as 13–48% among the six founders (Table 1 R2Ol-r100-v). Previous studies of DNA transposon in zebrafish indicated the germline mosaicism of 0.4–16% for Sleeping Beauty and 50% for Tol2, suggesting R2Ol showed sufficiently high germline transmission efficiency comparable to DNA transposons (Additional file 4: Table S3).

**Table 1:** R2Ol retrotransposed into the zebrafish 28S rDNA and transmitted to next generation

mRNA constructs	F0 retrotrans-position	Founder number	Founder (F0)	Germline mosaicism			Insertion copies	F1 name	F2 GFP+
				adult (fin)	embryo	total			
R2Ol-r100-v	53/55 (96%)	8/16 (50%)	R2f-2	8/25	4/30	12/55 (22%)	3	–	–
			R2f-5	–	4/30	4/30 (13%)	–	–	–
			R2f-6	2/8	–	2/8 (25%)	–	–	–
			R2f-7	4/33	5/30	9/63 (14%)	–	–	–
			R2f-9	2/10	–	2/10 (20%)	2	–	–
			R2f-10	2/3	18/39	20/42 (48%)	1	–	–
			R2f-11	–	–	–	–	–	–
			R2f-12	–	–	–	–	–	–
r105-R2Ol-EF1-EGFP(F)-r10	17/20 (85%)	2/27 (7.4%)	GFm-6 (truncated)	–	–	–	–	–	–
			GFm-13 (truncated)	–	–	–	–	–	–
r105-R2Ol-EF1-EGFP(R)-r10	21/22 (95%)	4/25 (16%)	GRm-1	1/42	5/30	6/72 (8.3%)	1	GRm-1-32	
			GRm-12 (truncated)	–	–	–	–	–	–
			GRm-14	3/7	17/30	20/37 (54%)	1	GRm-14-1	1/51
								GRm-14-7	2/124, 1/66
			GRf-2	3/21	2/30	5/51 (9.8%)	1	–	–
r105-R2Ol-hsp-GAL4R-r10	36/58 (62%)	1/15 (6.6%)	G4Rf-201	5/11	8/30	13/41 (32%)	1	G4Rf-201	15/29
R2OlΔ3′ UTR-v + EGFP(R)-3′ UTR-r10	3/44 (6.8%)	–	–	–	–	–	–	–	–

The retrotransposition efficiency (%) = transposed embryo no. / analyzed embryo no. × 100

### Transgene is integrated into 28S rDNA

**Fig. 3:**
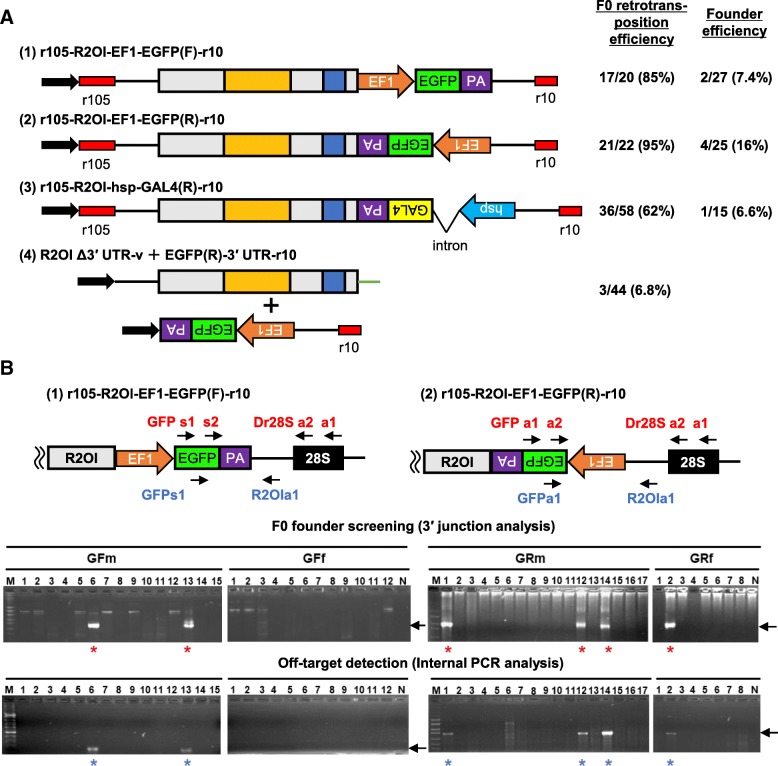
The R2Ol system generates transgenic zebrafish. a (1)–(3) Schematic diagram of R2Ol with transgene cassettes used in the retrotransposition assay. EGFP cassette includes EF1, Xenopus EF1-alpha promoter; EGFP, enhanced green fluorescent protein (EGFP) ORF forward or reverse orientations; PA, SV40 poly(A) signal; GAL4 cassette includes heat shock promoter (hsp), and GAL4 transcription factor. All constructs contain a 105-bp 28S rDNA sequence upstream of the 5′ UTR to increase full-length insertion and a 10-bp 28S rDNA sequence downstream of the 3′ UTR. (4) Two constructs for the trans-complementation assay. F0 retrotransposition efficiency and founder efficiency for each construct are indicated on the right (see also Table 1). **b** F0 founder screening and off target detection. Top, PCR founder screening of the 3′ junction region using pooled F1 genomic DNA. Primers are indicated by arrows. Bottom, internal PCR to detect external 28S rDNA insertions. Red asterisks indicate the obtained founders used in further analyses. Blue asterisks indicate internal PCR results

**Fig. 4:**
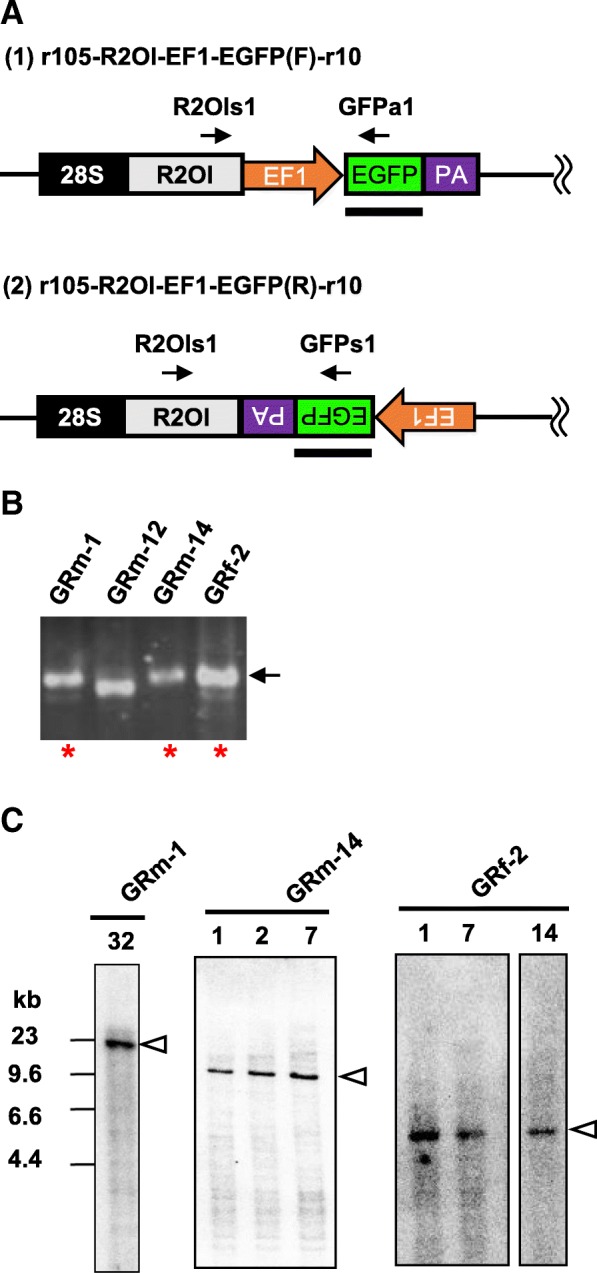
PCR analysis of the end of EGFP cassette integrated by r105-R2Ol-EF1-EGFP(R)-r10 injection. **a** Detection of 5′ end EGFP cassette. **b** Three founder, GRm-1, GRm-14 and GRf-2 produced R2Ol-EGFP junction fragments (red asterisks). GRm-12 fragment was shorter than expected size (black arrow) and sequencing revealed that it was nonspecific sequence. **c** Transgenic progeny analysis from three R2Ol-EGFP(R) founders, GRm-1, GRm-14, and GRf-2. Genomic DNA were digested with PvuII

Next, to achieve sequence-specific gene integration using R2Ol, we introduced an enhanced green fluorescent protein (EGFP) cassette that included the Xenopus elongation factor 1-alpha (EF1-alpha) promoter, EGFP ORF, and an SV40 poly(A) signal between the R2Ol ORF and 3′ UTR in both the forward and reverse orientations (Fig. 3a, (1), and (2)). The constructs exhibited F0 retrotransposition efficiency rates of 85 and 95%, respectively, and all inserted precisely into the 28S rDNA target site. After screening the F0 fish, two founders with forward-oriented EGFP, GFm-6 and -13, as well as four founders with reverse-oriented EGFP, GRm-1, − 12, − 14, and GRf-2 were identified (Fig. 3b, top panel, Table 1). Among these six founders, three (GRm-1, 14, and GRf-2) harbored complete EGFP cassettes according to PCR analysis (Fig. 4 a, b). Southern blot analysis revealed that only a single transgene insertion had occurred in each line (Fig. 4c). The germline mosaicism rates in the founders GRm-1, 14, and GRf-2 were 8.3, 54, and 9.8%, respectively (Table 1). These results indicated that accurate and efficient retrotransposition activity without off-target insertion was retained, even though the transgene (full-length EGFP cassette) was introduced into R2Ol and efficiently transmitted in germ cells. To further show that off-target insertion did not occur, we amplified both the 3′-junction region of R2Ol-28S rDNA and an internal region of the EGFP cassette via PCR from DNA of individual that was used for founder screening (Fig. 3b, bottom panel). Using this internal region PCR, it was possible to detect cassette insertion even outside 28S rDNA. We detected an internal region PCR band only from the genomic DNA sample in which we had detected the 3′-junction PCR band representing the R2Ol insertion. In conclusion, all the above results of Southern blot hybridization and internal region PCR assays indicate that R2Ol insertion did not occur outside the 28S rDNA. We further tested trans-complementation in the R2Ol system using the ability of R2Ol recognizing its 3′ UTR in the initial step of TPRT. When mRNAs from an R2Ol helper plasmid without 3′ UTR (Fig. 3a, (4) R2OlΔ3′ UTR-v) and a reporter plasmid of EGFP fused with R2Ol 3′ UTR (Fig. 3a, (4) EGFP(R)-3′ UTR-r10) were co-injected, we analyzed the retrotransposition activity of the EGFP construct. Of 44 embryos tested, we detected the precise retrotransposition event in three samples. This result of trans-complementation in the R2Ol system indicates easier manipulation and designing of the transgene construct, although the efficiency should be improved in future studies (Table 1).

### Transgene is expressed in zebrafish

**Fig. 5:**
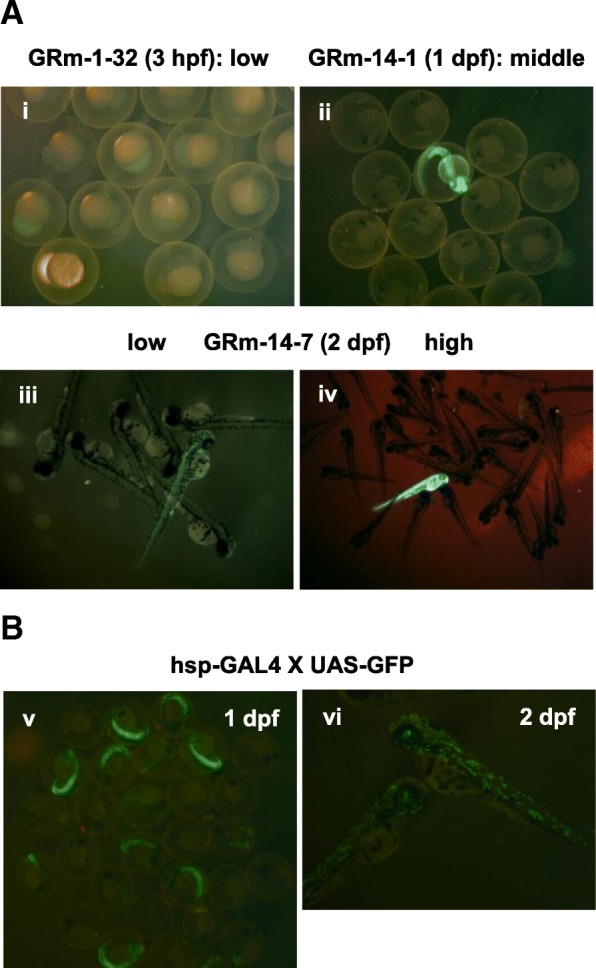
The transgenic EGFP is expressed in zebrafish. **a** EGFP expression pattern of the F2 progeny of two R2Ol–EGFP(R) transgenic lines. GRm-1-32 (i), GFm-14-1(ii) and − 14-7 (iii and iv) F1 females were mated with WT. hpf, hours post-fertilization. Dpf, days post-fertilization. Low, middle and high indicate the EGFP expression level. **b** Heat-shocked F2 progeny of a cross between the R2Ol–GAL4-R (heterozygous) and UAS-GFP lines (homozygous). dpf, days post-fertilization. Each image was taken from an independent mating

To evaluate the expression of the transgene inserted into the rDNA target, we obtained F2 progeny embryos from three transgenic F1 fish (GRm-1, GRm-14, and GRf-2) harboring a full-length EGFP cassette in reverse orientation (Additional file 1: Figure S3-iv). EGFP, following reverse-oriented insertion into rDNA, is expected to be transcribed by the polymerase II (pol II) from promoter, EF1-alpha. Although not all, several F2 embryos from a cross between a transgenic F1 and WT exhibited EGFP signals, with the expression levels varying among individuals (Fig, 5a-i, ii, iii, iv and Table 1). The EGFP signals were observed during the earlier stages of embryogenesis and decayed gradually with development. The silencing of pol II-dependent transgene transcription (through the Xenopus EF1-alpha promoter) within an rDNA tandem array or during translational processes may explain these phenomena, although further investigation is necessary to elucidate this mechanism. We also introduced a heat shock protein (hsp)-GAL4 cassette that expressed GAL4 from a heat shock promoter (Fig. 3a, (3)). One founder harboring a full-length hsp-GAL4 cassette in reverse orientation was obtained from 15 injected fishes, and five F1 progenies harbored the same single insertion (Additional file 1: Figure S5 A,B; Table 1). The founder was mated with the UAS-GFP line, and EGFP signals were observed in 52% F2 embryos obtained from a cross between the hsp-GAL4 (heterozygous) and UAS-GFP lines (homozygous) following heat shock administration (Fig. 5b-v, vi; Additional file 1: Figure S5C; Table 1). These results indicated that the hsp promoter was functional in an rDNA array of all GAL4-positive fish. Because the GAL4 cassette contains an intron between the promoter and ORF, a possible explanation for this increase in GAL4-expressing progeny is nuclear transportation for splicing.

## Discussion

Transposable elements have been widely used for a long time for genome editing technologies which are recently applied to vertebrates. For instance, to generate insertional mutagenesis, DNA transposons and L1 have been successfully used in various vertebrate models [22–25]. Especially the Tol2 transposon system which has contributed greatly to zebrafish research, as exemplified by the efficient germ line transmission and creation of transgenic zebrafish [26, 27], transfer of large exogenous genes [28], and generation of the Gal4-UAS system [29]. On the other hand, there are some approaches to develop the target-specific integration system using transposable elements. When transposase encoded in DNA transposons, Sleeping Beauty or piggyBac, are fused with DNA binding domains (DBDs) such as zinc fingers (ZFs) and TAL effectors, those constructs showed altered or biased targeting preference to specific sites [30, 31]. Additionally, group II intron vectors (targetrons) have been used for sequence-specific transgenesis across a wide range of bacteria [32].

In this study, we isolated an active form of sequence-specific LINE R2Ol from vertebrates and established for the first time an effective and highly sequence-specific retrotransposition system of R2Ol in zebrafish. This system has two potential advantages over the CRISPR/Cas9 system. Firstly, R2Ol does not require HDR and donor DNA. Instead, the target genes of interest are inserted via TPRT using an mRNA template. The most distinctive characteristic of TPRT is the ability of R2 RT to use the 3′ DNA end to prime reverse transcription. The RT of R2 elements synthesize cDNAs with high processivity and fidelity, which is advantageous for synthesizing long cDNAs onto the target site rather than recombination into the target site as donor DNA [21]. Secondly, R2Ol inserts site-specifically into 28S rDNA, which could be a candidate target site for gene integration. To date, only three intragenic sites (AAVS1, CCR5, and ROSA26) have been validated as genomic safe harbours (GSHs) in the human genome for the integration of therapeutic vectors [33]. However, they are located in gene-rich regions that are especially near the cancer-causing genes. In most vertebrates, it is known that several hundred rDNA copies exist in chromosomes, which means that multiple transgenes could be integrated simultaneously or additionally into the rDNA target without adversely affecting endogenous gene structure or expression. Several former studies have reported transgene integration into rDNA targets, but high target specificity was not achieved in most methods [34–37]. In this study, transgene-fused R2Ol maintained high retrotransposition activity and was efficiently transmitted to zebrafish germ cells, as well as off-target integration was not observed.

Although R2Ol has been shown to retrotranspose in zebrafish and be transmitted to future generations through the germ line, two problems should be solved before using it as an effective transgenic tool. The first problem is 5’UTR truncation, is often observed in endogenous LINEs, and this may disturb the insertion of longer genes. Addition of longer upstream 28S rDNA sequence to the 5′ end of the injected RNA may elongate the reverse transcript, as shown in R2Bm element [20]. Secondly, we should think about using a more appropriate promoter to express the transgene integrated in the rDNA array, because some results in this study often showed silencing of the integrated gene. Further study using various promoters and other reporter genes is needed to clarify how the transgene is expressed in the rDNA tandem array, which will help to establish the R2Ol system as a more practical and widely applicable transgenesis tool.

## Conclusions

In this study, we isolated R2Ol from medaka fish (*Oryzias latipes*) and demonstrated its sequence-specific retrotransposition in a distantly related zebrafish (*Danio rerio*). Particularly, the retrotransposition efficiency reached nearly 100% with several constructs, and the germline transmission and mosaicism rate was also high. Transgene-fused R2Ol maintained a high retrotransposition activity and was efficiently transmitted to zebrafish germ cells without off-target integration. These results indicate that R2Ol has the high and accurate retrotransposition activity in the 28S rDNA target site, which may provide an alternative and effective targeted gene knockin method in vertebrates.

## Methods

### Medaka fish

Medaka fish strain Hd-rR [38] was a kind gift from Drs. S. Oda and H. Mitani.

### Zebrafish

The strains of zebrafish (*Danio rerio*) were maintained at 28.5 °C in a 14-h light/10-h dark cycle as described by [39]. WIK [40], TL [41], and RIKEN WT (RIKEN) each for microinjection and for mating with transgenic fish of WIK, respectively. TAB and UAS-GFP transgenic fish was also kindly gifted by Prof. Kawakami. All animal protocols were approved by the University of Tokyo animal care and use committee (Approval number C-14-1).

### Cloning of R2Ol

We previously cloned and sequenced the 3′ end fragment of R2Ol (GenBank accession numbers AB201410). In this study, we first amplified the R2Ol 5′ end fragment by PCR from medaka fish strain Hd-rR genomic DNA. The obtained fragments were cloned and sequenced, and a BLAST search was performed that recognized the fragment as a homolog of zebrafish R2, R2Dr (AB097126) which we had previously identified. Based on this sequence, full length R2Ol was amplified by PCR from medaka fish genomic DNA by primers R2Ol5′end and R2Ol3′end. A 4.2 kb amplified fragment was cloned and sequenced, and nine clones were identified (Additional file 1: Figure S2). Compared with the consensus sequence, all clones contained ≥1 non-synonymous mutation. One of the less frequent mutations, clone F, was named pGEM-R2Ol-F, and used for the R2Ol retrotransposition assay. All primers used in this study are listed in Additional file 2: Table S1.

### Plasmids

To create R2Ol in vitro transcription template vector pR2Olr4, a full-length R2Ol fragment from pGEM-R2Ol-F digested with *Eco*RI and *Spe*I, was inserted into *Eco*RI and *Spe*I sites and an SV40 poly(A) signal was inserted between *Not*I and *Sac*I sites in pBluescript II KS(+). An RT mutant was constructed by introducing a point mutation via inverse PCR from pR2Olr4 using primers R2Ol-RT(D → V) s and R2Ol-RT(D → V), and self-ligated to obtain pR2Ol-RTmt. An EN mutant was similarly constructed via inverse PCR from pR2Olr4 using primers R2Ol-EN(D → A) s and R2Ol-EN(D → A) as and self-ligated to obtain pR2Ol-ENmt. To construct R2OlΔ3′ UTR-v, pR2Olr4 was digested with *Not*I, blunted with Klenow Fragment (TaKaRa) to delete the *Not*I site, and self-ligated to obtain pR2OlΔNotI. A new *Not*I recognition site was inserted between the R2Ol ORF and 3′ UTR by inverse PCR from pR2OlΔNotI using primers NotI-s and NotI-as, *Not*I digestion, and self-ligation to obtain pR2OlNotI3′ UTR. This was digested with *Not*I and *Spe*I, blunted, and self-ligated to obtain R2OlΔ3′ UTR-v.The EGFP cassette plasmids were constructed by inserting *Not*I recognition site between the R2Ol ORF and 3′ UTR by inverse PCR from pR2Olr4 using primers NotI-s and NotI-as, *Not*I digestion, and self-ligation to obtain pR2OlNotI. A 105 bp 28S rDNA sequence was inserted between *Hin*dIII and *Eco*RI recognition sites upstream the 5′ UTR of pR2OlNotI. *Not*I, *Nde*I, and *Sph*I recognition sites were introduced via linker ligation into the *Not*I site of pR2OlNotI to obtain pR2OlNNS. *Sph*I, *Nsi*I, *Nhe*I, *Mlu*I, *Bcl*I, and *Bgl*II recognition sites were inserted by linker ligation into the *Sph*I site, to obtain R2OlNNMBB. The EF1α-EGFP cassette was kindly supplied by Prof. K. Kawakami (National Institute of Genetics) as pT2KXIGDin. Because the original EF1α-EGFP cassette contained an intron between the promoter and ORF, we first deleted the intron from pT2KXIGDin by *Bam*HI and *Sal*I, digestion, blunting, and self-ligated to obtain pKEF1G. An intronless EF1α-EGFP cassette was isolated by digesting *Xho*I and *Not*I from pKEF1G. Adenine was added by Ex taq incubation followed by TA-cloning to pGEM-T EASY (Invitrogen) in both orientations, resulting in pEF1GF and pEF1GR. EF1α-EGFP was isolated by digesting *Nde*I and *Sph*I from pEF1GR and inserted between the *Nde*I and *Sph*I sites of R2OlNNS to obtain pR2OlEGFPF. EF1α-EGFP was PCR amplified from pEF1GR using primers BglII-EF1p-F and BglII-polyA-R, digested with *Bgl*II, and inserted at the *Bgl*II site of R2OlNNMBB to obtain pR2OlEGFPR. EGFP(R)-3′ UTR-r10 was constructed by amplification of the R2Ol 3′ UTR portion and then sub-cloned into the *Not*I site in pBluescript II KS(+)-poly(A). Then, EF1α EGFP was digested from pKEF1G by *Xho*Iと*Bam*HI and inserted into the *Xho*Iと*Bam*HI sites 3′ UTR-r10. The hsp-GAL4 cassette was PCR amplified from pT2KhspGFF (kindly given by Prof. Kawakami) using primers BglII-hsp-F and MluI-PA (GFF), digested with *Bgl*II and *Mlu*I, and inserted between *Bgl*II and *Mlu*I sites of pR2OlNNMBB to obtain pR2OlGAL4(R).

### In vitro transcription

The template DNA for in vitro transcription was amplified by PCR. R2Ol-r4-v, R2Ol RTmut, and R2Ol ENmut were PCR amplified from pR2Olr4, pR2Ol-RTmt, and pR2Ol-ENmt, respectively. The primers used were T7 and T3. R2Ol-r4 was PCR amplified using primers T7 and R2Ol3′end+r4 from pR2Olr4. r105-R2Ol-EF1-EGFP(F)-r10, r105-R2Ol-EF1-EGFP(R)-r10, and r105-R2Ol-hsp-GAL4(R)-r10 were PCR amplified using primers T7 and R2Ol3′end+r10 from pR2OlEF1EGFP(F), pR2OlEF1EGFP(R), and pR2OlGAL4(R), respectively. The PCR products were purified using a PCR cleanup kit (Sigma) and used for 5′ capped mRNA synthesis in vitro transcription using MEGAscript (Ambion) with a cap analog according to manufacturer’s instructions. The synthesized mRNA was precipitated with lithium chloride.

### Retrotransposition assay in zebrafish

In vitro transcribed mRNA (25 ng/μl) with KCl (100 mM) and rohdamin (0.0125%) or phenol red (0.05%) were injected into zebrafish one-cell embryos. After 24 h, genomic DNA was extracted from each embryo using a DNA extraction buffer (10 mM Tris-HCl, 10 mM EDTA and 100 μg/ml proteinase K) for at least 3 h at 55 °C, and heating at 95 °C for 10 min. A 1/10 dilution was used as the PCR template. The 3′ junction of the R2Ol insertions was detected via nested PCR to identify retrotransposition events using primer R2Ol s1 and Dr28S a1 for 1st round PCR and R2Ol s2 and Dr28S a2 for 2nd round PCR. Both rounds were 35 cycles of 98 °C for 20 s, 60 °C for 30 s, and 72 °C for 30 s (Fig. 1a). The PCR products were directly sequenced to detect the 3′ junction sequences. The retrotransposition efficiency (%) = transposed embryo no. / analyzed embryo no. × 100. The 5′ junction of the R2Ol insertions was detected via nested PCR at three R2Ol positions. The forward primers were specific to an upstream sequence of the 28S rDNA target site, Dr28S s1 for 1st round PCR and Dr28S s2 for 2nd round PCR. The reverse primers were specific to three R2Ol positions: R2Ol a11 for 1st round PCR and R2Ol a12 for 2nd round PCR; R2Ol a21 for 1st round PCR and R2Ol a22 for 2nd round PCR; or R2Ol a31 for 1st round PCR and R2Ol a32 for 2nd round PCR. All rounds were 35 cycles of 98 °C for 20 s, 60 °C for 30 s, and 72 °C for 30 s. The PCR products were directly sequenced or TA-cloned and sequenced. To detect the retrotransposition events of r105-R2Ol-EF1-EGFP(F)-r10, PCR forward primers were used as follows: GFP s1 for 1st round PCR and GFP s2 for 2nd round PCR. To detect the retrotransposition events of r105-R2Ol-EF1-EGFP(R)-r10 and trans-complementation retrotransposition events (R2OlΔ3′ UTR-v + EGFP(R)-3′ UTR-r10), the following PCR forward primers were used: GFP a1 for 1st round PCR and GFP a2 for 2nd round PCR. To detect the retrotransposition events of r105-R2Ol-hsp-GAL4(R)-r10, PCR forward primers were used as follow: hsp414-R for 1st round PCR and hsp154-R for 2nd round PCR.

### Founder fish screening

The injected fish were grown to adulthood and mated with WT to gain F1 embryos. Genomic DNA was extracted from at least 100 pooled embryo, and 3′ junction PCR was performed to screen for R2Ol founder.

### Transgenic fish screening

Genomic DNA was extracted from clipped caudal fins by incubating in 400 μl DNA extraction buffer (10 mM Tris-HCl, 10 mM EDTA and 100 μg/ml proteinase K) at 55 °C for more than 3 h. The DNA was purified by phenol-chloroform extraction, then precipitated with 1 ml 100% ethanol, rinsed once with 70% ethanol, and suspend in 50 μl TE buffer. PCR of the 3′ junction region was conducted to screen for R2Ol or transgene positive fish, followed by Southern blot.

### Southern blot hybridization

Each fish caudal fin genomic DNA (5 μg) was digested with appropriate restriction enzymes, run on a 1% agarose gel, and transferred to a membrane (Pole), hybridized with ^32^P-labeled probes. Radioactive signals were detected utilizing imaging plates and a BAS-5000 scanner (Fujifilm). Probe templates specific to full-length R2Ol, EGFP, or GAL4 were amplified by PCR and radiolabeled using BcaBEST (TaKaRa).

### Heat shock

Embryos obtained from mating hsp-GAL4 with UAS-GFP at 1 day post fertilization (dpf) were incubated at 37 °C for 1 h, 3 h, or 6 h on a heat block. Following the heat-shock treatment, the embryos were observed by microscope to detect EGFP expression.

## Additional files

Additional file 1: **Figure S1.** Comparison of R2 element insertion site. **Figure S2.** Nine clones of R2Ol obtained from medaka fish genomic DNA. **Figure S3.** A scheme for transgenesis in zebrafish using R2Ol. **Figure S4.** F1 progeny analysis of two founder lines, R2f-9 and R2f-10. **Figure S5.** Experimental approach for analysis of hsp-GAL4:UAS-EGFP expression induced by heat shock. (PDF 836 kb)

Additional file 2: **Table S1.** Primer List. (PDF 195 kb)

Additional file 3: **Table S2.** 5' junction sequence of inserted R2Ol. (PDF 59 kb)

Additional file 4: **Table S3.** Previously reported performance of representative DNA transposons in vertebrate. (PDF 116 kb)

## Abbreviations

bp: Base pair
CRISPR/Cas9: Clustered regularly interspaced short palindromic repeats / CRISPR associated proteins
dCas9: Dead Cas9 (Cas9 Endonuclease Dead)
dpf: Day post fertilization
DSBs: Double-strand breaks
EF1-alpha: Xenopus elongation factor 1-alpha promoter
EGFP: Enhanced green fluorescent protein
EN: Endonuclease domain
ENmut: Endonuclease domain mutant
F0 fish: Founder 0 fish
GSHs: Genomic safe harbours
h: Hours
HDR: Homology directed repair
hpf: Hours post-fertilization (hsp)-GAL4 heat shock protein (hsp)-GAL4(galactose-induced genes)
L1: Long interspersed element 1
LINEs: Long interspersed elements
LTR: Long terminal repeat
NHEJ: Non-homologous end-joining
ORF: Open reading frame
PCR: Polymerase chain reaction
pol II: Polymerase II promoter;
R2Ol: R2 from *Oryzias latipes*
r4: 4-bp 28S rDNA flanking sequence
rDNA: ribosomal DNA
RNP: Ribonucleoprotein
RT: Reverse transcriptase
RTmut: Reverse transcriptase mutant
SV40 poly-A signal: Simian Vacuolating virus 40 poly-A signal
TALE: Transcription activator like effector
TPRT: Target-primed reverse transcription
UAS-GFP: Upstream Activation Sequence
UTR: Untranslated region
v: Vector sequence
WT: Wild type
ZFs: (zinc fingers)
