# Supplementary material for: Targeted gene knockin in zebrafish using the 28S rDNA-specific non-LTR-retrotransposon R2Ol
Source: Mob DNA. 2019 May 22;10:23. doi: 10.1186/s13100-019-0167-2 (PMC6530143; doi:10.1186/s13100-019-0167-2)
Supplement: Supplementary file 1 — Figure S1. Comparison of R2 element insertion site. Figure S2. Nine clones of R2Ol obtained from medaka fish genomic DNA. Figure S3. A scheme for transgenesis in zebrafish using R2Ol. Figure S4. F1 progeny analysis of two founder lines, R2f-9 and R2f-10. Figure S5. Experimental approach for analysis of hsp-GAL4:UAS-EGFP expression induced by heat shock. (PDF 836 kb) [file 13100_2019_167_MOESM1_ESM.pdf]

Additional file 1:

Targeted gene knockin in zebrafish using the 28S rDNA-specific non-LTR-retrotransposon R2Ol

Azusa Kuroki-Kami[1,3], Narisu Nichuguti[1,3], Haruka Yatabe[1], Sayaka Mizuno[1], Shoji Kawamura[2], and Haruhiko Fujiwara[1,*]

[1] Department of Integrated Biosciences, Graduate School of Frontier Sciences, The University of Tokyo, Bioscience Bldg. 501, Kashiwanoha 5-1-5, Kashiwa, Chiba, 277-8562, Japan.
[2] Department of Integrated Biosciences, Graduate School of Frontier Sciences, The University of Tokyo, Bioscience Bldg. 502, Kashiwanoha 5-1-5, Kashiwa, Chiba, 277-8562, Japan.
[3] These authors contributed equally to this work.

* To whom correspondence should be addressed. Tel: +81-4-7136-3659; Fax: +81-4-7136-3660; Email: haruh@edu.k.u-tokyo.ac.jp

Additional file 1:

Figure S1. Comparison of R2 element insertion site.
Figure S2. Nine clones of R2Ol obtained from medaka fish genomic DNA.
Figure S3. A scheme for transgenesis in zebrafish using R2Ol.
Figure S4. F1 progeny analysis of two founder lines, R2f-9 and R2f-10.
Figure S5. Experimental approach for analysis of hsp-GAL4:UAS-EGFP expression induced by heat shock.

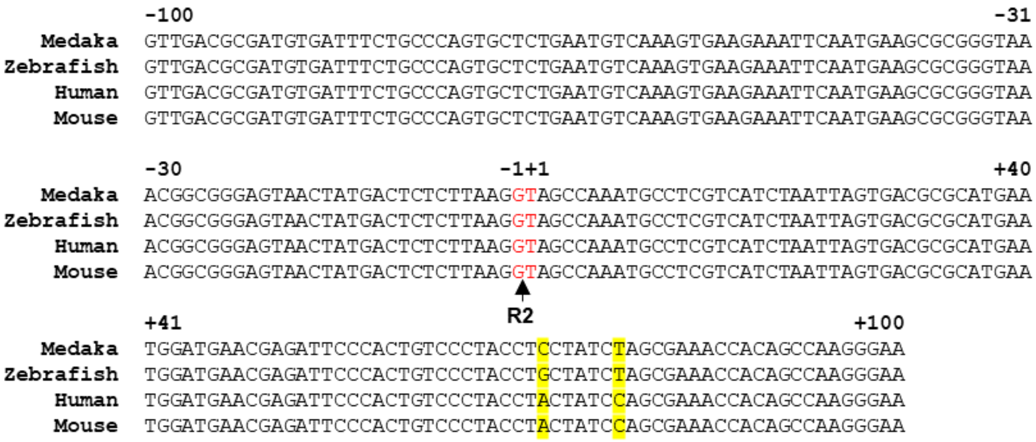

Figure S1. Comparison of R2 element insertion site. R2 insertion site sequence alignment of four vertebrates, medaka fish(CP020779.1), zebrafish(BX537263.17), human (M11167.1) and mouse (X00525.1). An approximately 200 base pair (bp) region around the R2 insertion site in 28S rDNA is strongly conserved except for differences at two nucleotides (shaded in yellow). The insertion site (red) of R2 is between -1 (G) and +1(T).

A

```
        1                                                                                                      100
CloneA  MGTDTVYVGQ DYPSGLSKRV PARLVAGPML RERSCHAHVF RAGHMWNWRT SLPSGRWDQP ALEKSRVLTR SVATATDPEI TSYPGKSVST STQVQEEDWC
CloneB  MGTDTVYVGQ DYPSGLSKRV PARLVAGPML RERSCHAHVF RAGHMWNWRT SLPSGRWDQP ALEKSRVLTR SVATATDPEI TSYPGKSVST STQVQEEDWC
CloneC  MGTDTVYVGQ DYPSGLSKRV PARLVAGPML RERSCHAHVF RAGHMWNWRT SLPSGRWDQP ALEKSRVLTR SVATATDPEI TSYPGKSVST STQVQEEDWC
CloneD  MGTDTVYVGQ DYPSGLSKRV PARLVAGPML RERSCHAHVF RAGHMWNWRT SLPSGRWDQP ALEKSRVLTR SVATATDPEI TSYPGKSVST STQVQEEDWC
CloneE  MGTDTVYVGQ DYPSGLSKRV PARLVAGPML RERSCHAHVF RAGHMWNWRT SLPSGRWDQP ALEKSRVLTR SVATATDPEI TSYPGKSVST STQVQEEDWC
CloneF  MGTDTVYVGQ DYPSGLSKRV PARLVAGPML RERSCHAHVF RAGHMWNWRT SLPSGRWDQP ALEKSRVLTR SVATATDPEI TSYPGKSVST STQVQEEDWC
CloneG  MGTDTVYVGQ DYPSGLSKRV PARLVAGPML RERSCHAHVF RAGHMWNWRT SLPSGRWDQP ALEKSRVLTR SVATATDPEI TSYPGKSVST STQVQEEDWC
CloneI  MGTDTVYVGQ DYPSGLSKRV PARLVAGPML RERSCHAHVF RAGHMWNWRT SLPSGRWDQP ALEKSRVLTR SVATATDPEI TSYPGKSVST STQVQEEDWC
CloneJ  MGTDTVYVGQ DYPSGLSKRV PARLVAGPML RERSCHAHVF RAGHMWNWRT SLPSGRWDQP ALEKSRVLTR SVATATDPEI TSYPGKSVST STQVQEEDWC

        101                                                                                                    200
CloneA  SRESGWISPG LAPEEPSVVS EITASMVATM RVATEEVVLE PQPEQVVTIL PGHGRNVPPG LAEQDTASPI EVSVLLPDLA ENCPLCGVPS GGLRLLGKHF
CloneB  SRESGWISPG LAPEEPSVVS EITASMVATM RVATEEVVLE PQPEQVVTIL PEHGRNVPPG LAEQDTASPI EVSVLLPDLA ENCPLCGVPS GGLRLLGKHF
CloneC  SRESGWISPG LAPEEPSVVS EITASMVATM RVATEEVVLE PQPEQVVTIL PEHGRNVPPG LAEQDTASPI EVSVLLPDLA ENCPLCGVPS GGLRLLGKHF
CloneD  SRESGWISPG LAPEEPSVVS EITASMVATM RVATEEVVLE PQPEQVVTIL PEHGRNVPPG LAEQDTASPI EVSVLLPDLA ENCPLCGVPS GGLRLLGKHF
CloneE  SRESGWISPG LAPEEPSVVS EITASMVATM RVATEEVVLE PQPEQVVTIL PEHGRNVPPG LAEQDTASPI EVSVLLPDLA ENCPLCGVPS GGLRLLGKHF
CloneF  SRESGWISPG LAPEEPSVVS EITASMVATM RVATEEVVLE PQPEQVVTIL PEHGRNVPPG LAEQDTASPI EVSVLLPDLA ENCPLCGVPS GGLRLLGKHF
CloneG  SRESGWISPG LAPEEPSVVS EITASMVATM RVATEEVVLE PQPEQVVTIL PEHGRNVPPG LAEQDTASPI EVSVLLPDLA ENCPLCGVPS GGLRLLGKHF
CloneI  SRESGWISPG LAPEEPSVVS EITASMVATM RVATEEVVLE PQPEQVVTIL PEHGRNVPPG LAEQDTASPI EVSVLLPDLA ENCPLCGVPS GGLRLLGKHF
CloneJ  SRESGWISPG LAPEEPSVVS EITASMVATM RVATEEVVLE PQPEQVVTIL PEHGRNVPPG LAEQDTASPV EVSVLLPDLA ENCPLCGVPS GGLRLLGKHF

        201                                                                                                    300
CloneA  AVRHAGVPVT YECRKCAWRS PNSHSISCHV PKCRGRARVP SGDPGIACDL CEARFATEVG VAQHKRHVHP VEWNKVRLER RGARGGGIKA TKLWSVAEVE
CloneB  AVRHAGVPVT YECRKCAWRS PNSHSISCHV PKCRGRARVP SGDPGIACDL CEARFATEVG VAQHKRHVHP VEWNKVRLER RGARGGGIKA TKLWSVAEVE
CloneC  AVRHAGVPVT YECRKCARRS PNSHSISCHV PKCRGRARVP SGDPGIACDL CEARFATEVG VAQHKRHVHP VEWNKVRLER RGARGGGIKA TKLWSVAEVE
CloneD  AVRHAGVPVT YECRKCAWRS PNSHSISCHV PKCRGHARVP SGDPGIACDL CEARFATEVG VAQHKRHVHP VEWNKVRLER RGARGGGIKA TKLWSVAEVE
CloneE  AVRHAGVPVT YECRKCAWRS PNSHSISCHV PKCRGRARVP SGDPGIACDL CEARFATEVG VAQHKRHVHP VEWNKVRLER RGARGGGIKA TKLWSVAEVE
CloneF  AVRHAGVPVT YECRKCAWRS PNSHSISCHV PKCRGRARVP SGDPGIACDL CEARFATEVG VAQHKRHVHP VEWNKVRLER RGARGGGIKA TKLWSVAEVE
CloneG  AVRHAGVPVT YECRKCAWRS PNSHSISCHV PKCRGRARMP SGDPGIACDL CEARFATEVG VAQHKRHVHP VEWNKVRLER RGARGGGIKA TKLWSVAEVE
CloneI  AVRHAGVPVT YECRKCAWRS PNSHSISCHV PKCRGRAQMP SGDPGIACDL CEARFATEVG VAQHKRHVHP VEWNKVRLER RGARGGGIKA TKLWSVAEVE
CloneJ  AVRHAGVPVT YECRKCAWRS PNSHSISCHV PKCRGRARVP SGDPGIACDL CEARFATEVG VAQHKRHVHP VEWNKVRLER RGARGGGIKA TKLWSVAEVE

        301                                                                                                    400
CloneA  TLIRLIREHG DSGATYQLIA DELGRGKTAE QVRSKKRLLR IDTASNSPDD AEVEEERLES LAVRSSSRSP PSLVATRVRE AVARGESEGG EEIRAIAALI
CloneB  TLIRLIREHG DSGATYQLIA DELGRGKTAE QVRSKKRLLR IDTASNSPDD AEVEEERLES LAVRSSSRSP PSLVATRVRE AVARGESEGG EEIRAIAALI
CloneC  TLIRLIREHG DSGATYQLIA DELGRGKTAE QVRSKKRLLR IDTASNSPDD AEVEEERLES LAVRSSSRSP PSLVATRVRE AVARGESEGG EEIRAIAALI
CloneD  TLIRLIREHG DSGATYQLIA DELGRGKTAE QVRSKKRLLR IDTASNSPDD AEVEEERLES LAVRSSSRSP PSLVATRVRE AVARGESEGG EEIRAIAALI
CloneE  TLIRLIREHG DSGATYQLIA DELGRGKTAE QVRSKKRLLR IDTASNSPDD AEVEEERLES LAVRSSSRSP PSLVATRVRE AVARGESEGG EEIRAIAALI
CloneF  TLIRLIREHG DSGATYQLIA DELGRGKTAE QVRSKKRLLR IDTASNSPDD AEVEEERLES LAVRSSSRSP PSLVATRVRE AVARGESEGG EEIRAIAALI
CloneG  TLIRLIREHG DSGATYQLIA DELGRGKTAE QVRSKKRLLR IDTASNSPDD AEVEEERLES LAVRSSSRSP PSLVATRVRE AVARGESEGG EEIRAIAALI
CloneI  TLIRLIREHG DSGATYQLIA DELGRGKTAE QVRSKKRLLR IDTASNSPDD AEVEEERLES LAVRSSSRSP PSLVATRVRE AVARGESEGG EEIRAIAALI
CloneJ  TLIRLIREHG DSGATYQLIA DELGRGKTAE QVRSKKRLLR IDTASNSPDD AEVEEERLES LAVRSSSRSP PSLVATRVRE AVARGESEGG EEIRAIAALI

        401                                                                                                    500
CloneA  RDVDQNPCLI ETSASDIISK LGRRVDGPKR PRPVVREQTQ EKGWVRRLAR RKREYREAQY LYSRDQARLA AQILDGAASQ ECALPVDQVY GAFREKWETV
CloneB  RDVDQNPCLI ETSASDIISK LGRRVDGPKR PRPVVREQTQ EKGWVRRLAR RKREYREAQY LYSRDQARLA AQILDGAASQ ECALPVDQVY GAFREKWETV
CloneC  RDVDQNPCLI ETSASDIISK LGRRVDGPKR PRPVVREQTQ EKGWVRRLAR RKREYREAQY LYSRDQARLA AQILDGAASQ ECALPVDQVY GAFREKWETV
CloneD  RDVDQNPCLI ETSASDIISK LGRRVDGPKR PRPVVREQTQ EKGWVRRLAR RKREYREAQY LYSRDQARLA AQILDGAASQ ECALPVDQVY GAFREKWETV
CloneE  RDVDQNPCLI ETSASDIISK LGRRVDGPKR PRPVVREQTQ EKGWVRRLAR RKREYREAQY LYSRDQARLA AQILDGAASQ ECALPVDQVY GAFREKWETV
CloneF  RDVDQNPCLI ETSASDIISK LGRRVDGPKR PRPVVREQTQ EKGWVRRLAR RKREYREAQY LYSRDQARLA AQILDGAASQ ECALPVDQVY GAFREKWETV
CloneG  RDVDQNPCLI ETSASDIISK LGRRVDGPKR PRPVVREQTQ EKGWVRRLAR RKREYREAQY LYSRDQARLA AQILDGAASQ ECALPVDQVY GAFREKWETV
CloneI  RDVDQNPCLI ETSASDIISK LGRRVDGPKR PRPVVREQTQ EKGWVRRLAR RKREYREAQY LYSRDQARLA AQILDGAASQ ECALPVDQVY GAFREKWETV
CloneJ  RDVDQNPCLI ETSASDIISK LGRRVDGPKR PRPVVREQTQ EKGWVRRLAR RKREYREAQY LYSRDQARLA AQILDGAASQ ECALPVDQVY GAFREKWETV

        501                                                                                                    600
CloneA  GQFHGLGEFR TGARADNWEF YSPILAAEVK ENLMRMANGT APGPDRISKK VLLDWDPRGE QLARLYTTWL IGGVIPRVFK ECRTKLLPKS SDPVELQDIG
CloneB  GQFHGLGEFR TGARADNWEF YSPILAAEVK ENLMRMANGT APGPDRISKK ALLDWDPRGE QLARLYTTWL IGGVIPRVFK ECRTKLLPKS SDPVELQDIG
CloneC  GQFHRLGEFR TGARADNWEF YSPILAAEVK ENLMRMANGT APGPGRISKK ALLDWDPRGE QLARLYTTWL IGGVIPRVFK ECRTKLLPKS SDPVELQDIG
CloneD  GQFHGLGEFR TGARADNWEF YSPILAAEVK ENLMRMANGT APGPDRISKK ALLDWDPRGE QLARLYTTWL IGGVIPRVFK ECRTKLLPKS SDPVELQDIG
CloneE  GQFHGLGEFR TGARADNWEF YSPILAAEVK ENLMRMANGT APGPDRISKK ALLDWDPRGE QLARLYTTWL IGGVIPRVFK ECRTKLLPKS SDPVELQDIG
CloneF  GQFHGLGEFR TGARADNWEF YSPILAAEVK ENLMRMANGT APGPDRISKK ALLDWDPRGE QLARLYTTWL IGGVIPRVFK ECRTKLLPKS SDPVELQDIG
CloneG  GQFHGLGEFR TGARADNWEF YSPILAAEVK ENLMRMANGT APGPDRISKK ALLDWDPRGE QLARLYTTWL IGGVIPRVFK ECRTKLLPKS SDPVELQDIG
CloneI  GQFHGLGEFR TGARADNWEF YSPILAAEVK ENLMRMANGT APGPDRISKK ALLDWDPRGE QLARLYTTWL IGGVIPRVFK ECRTKLLPKS SDPVELQDIG
CloneJ  GQFHGLGEFR TGARADNWEF YSPILAAEVK ENLMRMANGT APGPDRISKK ALLDWDPRGE QLARLYTTWL IGGVIPRVFK ECRTKLLPKS SDPVELQDIG
```

A continued

```
        601                                                                                                 700
CloneA  GWRPVTIGSM VTRLFSRILT MRLTRACPIN PRQRGFLASS SGCAENLLIF DEIVRRSRRD GGPLAVVFVD FARAFDSISH EHILCVLEEG GLDRHVIGLI
CloneB  GWRPVTIGSM VTRLFSRILT MRLTRACPIN PRQRGFLASS SGCAENLLIF DEIVRRSRRD GGPLAVVFVD FARAFDSISH EHILCVLEEG GLDRHVIGLI
CloneC  GWRPVTIGSM VTRLFSRILT MRLTRACPIN PRQRGFLASS SGCAENLLIF DEIVRRSRRD GGPLAVVFVD FARAFDSISH EHILCVLEEG GLDRHVIGLI
CloneD  GWRPVTIGSM VTRLFSRILT MRLTRACPIN PRQRGFLASS SGCAENLLIF DEIVRRSRRD GGPPAVVFVD FARAFDSISH EHILCVLEEG GLDRHVIGLI
CloneE  GWRPVTIGSM VTRLFSRILT MRLTRACPIN PRQRGFLASS SGCAENLLIF DEIVRRSRRD GGPLAVVFVD FARAFDSISH EHILCVLEEG GLDRHVIGLI
CloneF  GWRPVTIGSM VTRLFSRILT MRLTRACPIN PRQRGFLASS SGCAENLLIF DEIVRRSRRD GGPLAVVFVD FARAFDSISH EHILCVLEEG GLDRHVIGLI
CloneG  GWRPVTIGSM VTRLFSRILT MRLTRACPIN PRQRGFLASS SGCAENLLIF DEIVRRSRRD GGPLAVVFVD FARAFDSISH EHILCVLEEG GLDRHVIGLI
CloneI  GWRPVTIGSM VTRLFSRILT MRLTRACPIN PRQRGFLASS SGCAENLLIF DEIVRRSRRD GGPLAVVFVD FARAFDSISH EHILCVLEEG GLDRHVIGLI
CloneJ  GWRPVTIGSM VTRLFSRILT MRLTRACPIN PRQRGFLASS SGCAENLLIF DEIVRRSRRD GGPLAVVFVD FARAFDSISH EHILCVLEEG GLDRHVIGLI

       701                                                                                                 800
CloneA  RNSYVDCVTR VGCVEGMTPP IQMKVGVKQG DPMSPLLFNL AMDPLIHKLE TAGTGLKWGD LSIATLAFAD DLVLVSDSEE GMGRSLGILE KFCQLTGLRV
CloneB  RNSYVDCVTR VGCVEGMTPP IQMKVGVKQG DPMSPLLFNL AMDPLIHKLE TAGTGLKWGD LSIATLAFAD DLVLVSDSEE GMGRSLGILE KFCQLTGLRV
CloneC  RNSYVDCVTR VGCVEGMTPP IQMKVGVKQG DPMSPLLFNL AMDPLIHKLE TAGTGLKWGD LSIATLAFAD DLVLVSDSEE GMGRSLGILE KFCQLTGLRV
CloneD  RNSYVDCVTR VGCVEGMTPP IQMKVGVKQG DPMSPLLFNL AMDPLIHKLE TAGTGLKWGD LSIATLAFAD DLVLVSDSKE GMGRSLGILE KFCQLTGLRV
CloneE  RNSYVDCVTR VGCVEGMTPP IQMKVGVKQG DPMSPLLFNL AMDPLIHKLE TAGTGLKWGD LSIATLAFAD DLVLVSDSEE GMGRSLGILE KFCQLTGLRV
CloneF  RNSYVDCVTR VGCVEGMTPP IQMKVGVKQG DPMSPLLFNL AMDPLIHKLE TAGTGLKWGD LSIATLAFAD DLVLVSDSEE GMGRSLGILE KFCQLTGLRV
CloneG  RNSYVDCVTR VGCVEGMTPP IQMKVGVKQG DPMSPLLFNL AMNPLIHKLE TAGTGLKWGD LSIATLAFAD DLVLVSDSEE GMGRSLGILE KFCQLTGLRV
CloneI  RNSYVDCVTR VGCVEGMTPP IQMKVGVKQG DPMSPLLFNL AMDPLIHKLE TAGTGLKWGD LSIATLAFAD DLVLVSDSEE GMGRSLGILE KFCQLTGLRV
CloneJ  RNSYVDCVTR VGCVEGMTPP IQMKVGVKQG DPMSPLLFNL AMDPLIHKLE TAGTGLKWGD LSIATLAFAD DLVLVSDSEE GMGRSLGILE KFCQLTGLRV

        801                                                                                                 900
CloneA  QPRKCHGFFM DKGVVNGCGT WEICGSPIHM IPPGESVRYL GVQVGPGRGV MEPDLIPTVH TWIERISEAP LKPSQRMRVL NSFALPRIIY QADLGKVTVT
CloneB  QPRKCHGFFM DKGVVNGCGT WEICGSPIHM IPPGESVRYL GVQVGPGRGV MEPDLIPTVH TWIERISEAP LKPSQRMRVL NSFALPRIIY QADLGKVTVT
CloneC  QPRKCHGFFM DKGVVNGCGT WEICGSPIHM IPPGESVRYL GVQVGPGRGV MEPDLIPTVH TWIERISEAP LKPSQRMRVL NSFALPRIIY QADLGKVTVT
CloneD  QPRKCHGFFM DKGVVNGCGT WEICGSPIHM IPPGESVRYL GVQVGPGRGV MEPDLIPTVH TWIERISEAP LKPSQRMRVL NSFALPRIIY QADLGKVTVT
CloneE  QPRKCHGFFM DKGVVNGCGT WEICGSPIHM IPPGESVRYL GVQVGPGRGV MEPDLIPTVH TWIERISEAP LKPSQRMRVL NSFALPRIIY QADLGKVTVT
CloneF  QPRKCHGFFM DKGVVNGCGT WEICGSPIHM IPPGESVRYL GVQVGPGRGV MEPDLIPTVH TWIERISEAP LKPSQRMRVL NSFALPRIIY QADLGKVTVT
CloneG  QPRKCHGFFM DKGVVNGCGT WEICGSPIHM IPPGESVRYL GVQVGPGRGV MEPDLISTVH TWIERISEAP LKPSQRMRVL NSFALPRIIY QADLGKVTVT
CloneI  QPRKCHGFFM DKGVVNGCGT WEICGSPIHM IPPGESVRYL GVQVGPGRGV MEPDLIPTVH TWIERISEAP LKPSQRMRVL NSFALPRIIY QADLGKVTVT
CloneJ  QPRKCHGFFM DKGVVNGCGT WEICGSPIHM IPPGESVRYL GVQVGPGRGV MEPDLIPTVH TWIERISEAP LKPSQRMRVL NSFALPRIIY QADLGKVTVT

        901                                                                                                 1000
CloneA  KLAQIDGIVR KAVKKWLHLS PSTCNGLLYS RNRDGGLGLL KLERLIPSVR TKRIYRMSRS PDIWTRRMTS HSVSKSDWEM LWVQAGGERG SAPVMGAVEA
CloneB  KLAQIGGIVR KAVKKWLHLS PSTCNGLLYS RNRDGGLGLL KLERLIPSVR TKRIYRMSRS PDIWTRRMTS HSVSKSDWEM LWVQAGGERG SAPVMGAVEA
CloneC  KLAQIDGIVR KAVKKWLHLS PSTCNGLLYS RNRDGGLGLL KLERLIPSVR TKRIYRMSRS PDIWTRRMTS HSVSKSDWEM LWVQAGGERG SAPVMGAVEA
CloneD  KLAQIDGIVR KAVKKWLHLS PSTCNGLLYS RNRDGGLGLL KLERLIPSVR TKRIYRMSRS PDIWTRRMTS HSVSKSDWEM LWVQAGGERG SAPVMGAVEA
CloneE  KLAQIDGIVR KAVKKWLHLS PSTCNGLLYS RNRDGGLGLL KLERLIPSVR TKRIYRMSRS PDIWTRRMTS HSASKSDWEM LWVQAGGERG SAPVMGAVEA
CloneF  KLAQIDGIVR KAVKKWLHLS PSTCNGLLYS RNRDGGLGLL KLERLIPSVR TKRIYRMSRS PDIWTRRMTS HSVSKSDWEM LWVQAGGERG SAPVMGAVEA
CloneG  KLVQIDGIVR KAVKKWLHLS PSTCNGLLYS RNRDGGLGLL KLERLIPSVR TKRIYRMSRS PDIWTRRMTS HSVSKSDWEM LWVQAGGERG SAPVMGAVEA
CloneI  KLAQIDGIVR KAVKKWLHLS PSTCNGLLYS RNRDGGLGLL KLERLIPSVR TKRIYRMSRS PDIWTRRMTS HSASKSDWEM LWVQAGGERG SAPVMGAVEA
CloneJ  KLAQIDGIVR KAVKKWLHLS PSTCNGLLYS RNRDGGLGLP KLERLIPSVR TKRIYRMSRS PDIWTRRMTS HSVSKSDWEM LWVQAGGERG SAPVMGAVEA

        1001                                                                                                1100
CloneA  APTDVERSSD YPDWRREENL AWSALRVQGV GADQFRGDRT SSSWIAEPAS VGFAQRHWLA ALALRAGVYP TREFLARGKE KSGAACRRCP ARLESCSHIL
CloneB  APTDVERSPD YPDWRREENL AWSALRVQGV GADQFRGDRT SSSWIAEPAS VGFAQRHWLA ALALRAGVYP TREFLARGKE KSGAACRRCP ARLESCSHIL
CloneC  APTDVERTPD YPDWRREENL AWSALRVQGV GADQFRGDRT SSSWIAEPAS VGFAQRHWLA ALALRAGVYP TREFLARGKE KSGAACRRCP ARLESCSHIL
CloneD  APTDVERSPD YPDWRREENL AWSALRVQGV GADQFRGDRT SSSWIAEPAS VGFAQRHWLA ALALRAGVYP TREFLARGKE KSGAACRRCP ARLESCSHIL
CloneE  APTDVERSPD YPDWRREENL ARSALRVQGV GADQFRGDRT SSSWIAEPAS VGFAQRHWLA ALALRAGVYP TREFLARGKE KSGAACRRCP ARLESCSHIL
CloneF  APTDVERSPD YPDWRREENL AWSALRVQGV GADQFRGDRT SSSWIAEPAS VGFAQRHWLA ALALRAGVYP TREFLARGKE KSGAACRRCP ARLESCSHIL
CloneG  APTDVERSPD YPDWRREENL TWSALRVQGV GADQFRGDRT SSSWIAEPAS VGFAQRHWLA ALALRAGVYP TREFLARGKE KSGAACRRCP ARLESCSHIL
CloneI  APTDVERSPD YPDWRREENL ARSALRVQGV GADQFRGDRT SSSWIAEPAS VGFAQRHWLA ALALRAGVYP TREFLARGKE KSGAACRRCP ARLESCSHIL
CloneJ  APTDVERSPD YPDWRREENL AWSALRVQGV GADQFRGDRT SSSWIAEPAS VGFAQRHWLA ALALRAGVYP TREFLARGKE KSGAACRRCP ARLESCSHIL

        1101                                                                                                1200
CloneA  GQCPFVQANR IARHNKVCVL LATEAERFGW TVIREFRLED AAGGLKIPDL VCKKADTVLI VDVTVRYEMD GETLKRAASE KVEHYLPVGQ QITDKVGGRC
CloneB  GQCPFVQANR IARHNKVCVL LATEAERFGW TVIREFRLED AAGGLKIPDL VCKKADTVLI VDVTVRYEMD GETLKRAASE KVEHYLPVGQ QITDKVGGRC
CloneC  GQCPFVQANR IARHNKVCVL LATEAERFGW TVIREFRLED AAGGLKIPDL VCKKADTVLI VDVTVRYEMD GETLKRAASE KVEHYLPVGQ QITDKVGGRC
CloneD  GQCPFVQANR IARHNKVCVL LATEAERFGW TVIREFRLED AAGGLKIPDL VCKKADTVLI VDVTVRYEMD GETLKRAASE KVEHYLPVGQ QITDKVGGRC
CloneE  GQCPFVQANR IARHNKVCVL LATEAERFGW TVIREFRLED AAGGLKIPDL VCKKADTVLI VDVTVRYEMD GETLKRAASE KVEHYLPVGQ QITDKVGGRC
CloneF  GQCPFVQANR IARHNKVCVL LATEAERFGW TVIREFRLED AAGGLKIPDL VCKKADTVLI VDVTVRYEMD GETLKRAASE KVKHYLPVGQ QITDKVGGRC
CloneG  GQCPFVQANR IARHNKVCVL LATEAERFGW TVIREFRLED AAGGLKIPDL VCKKADTVLI VDVTVRYEMD GETLKRAASE KVEHYLPVGQ QITDKVGGRC
CloneI  GQCPFVQANR IARHNKVCVL LATEAERFGW TVIREFRLED AAGGLKIPDL VCKKADTVLI VDVTVRYEMD GETLKRAASE KVEHYLPVGQ QITDKVGGRC
CloneJ  GQCPFVQANR IARHNKVCVL LATEAERFGW TVIREFRLED AAGGLKIPDL VCKKADTVLI VDVTVRYEMD GETLKRAASE KVEHYLPVGQ QITDKVGGRC

        1201                                                                      1276
CloneA  FKVMGFPVDA RGKWPASNNT VLAELGVPAG RMRTFARLVS RRTLLYSLDI LRDFMREPAG RGTRVALIPA ATGAAN
CloneB  FKVMGFPVGA RGKWPASNNT VLAELGVPAG RMRTFARLVS RRTLLYSLDI LRDFMREPAG RGTRVALIPA ATGAAN
CloneC  FKVMGFPVGA RGKWPASNNT VLAELGVPAG RMRTFARLVS RRTLLYSLDI LRDFMREPAG RGTRVALIPA ATGAAN
CloneD  FKVMGFPVGA RGKWPASNNT VLAELGVPAG RMRTFARLVS RRTLLYSLDI LRDFMREPAG RGTRVALIPA ATGAAN
CloneE  FKVMGFPVGA RGKWPASNNT VLAELGVPAG RMRTFARLVS RRTLLYSLDI LRDFMREPAG RGTRVALIPA ATGAAN
CloneF  FKVMGFPVGA RGKWPASNNT VLAELGVPAG RMRTFARLVS RRTLLYSLDI LRDFMREPAG RGTRVALIPA ATGAAN
CloneG  FKVMGFPVGA RGKWPASNNT VLAELGVPAG RMRTFARLVS RRTLLYSLDI LRDFMREPAG RGTRVALIPA ATGAAN
CloneI  FKVMGFPVGA RGKWPASNNT VLAELGVPAG RMRTFARLVS RRTLLYSLDI LRDFMREPAG RGTRVALIPA ATGAAN
CloneJ  FKVMGFPVGA RGKWPASNNT VLAELGVPAG RMRTFARLVS RRTLPYSLDI LRDFMREPAG RGTRVALIPA ATGAAN
```

B

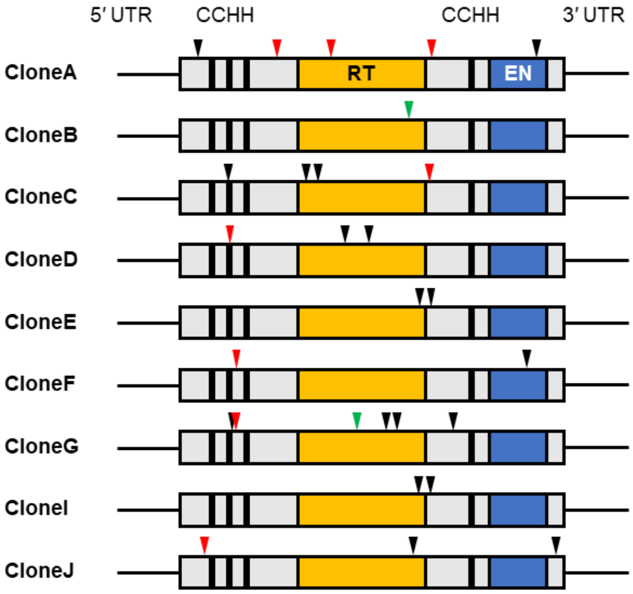

C

CGCACAGGGGACACAGAGCCTGCCCAAGTACCGCTCCCGAGGGAGCGGGAAACGGGGGGGTGACTATCCCCTGGGGTCCGGCGAGAGCGCTGGTCTACGGACCA
GGGGTGGCTGTGGGCAGGCTGCTCCTCAGGCCAGTTGATTAGTTACGCATGGGCTGTACCTCCACGTGGTCCCGCTGGTAACGACTTGTCGGCTAAATCAGCCC
GCCCACCATCTGGGATATGGTTGACCGTCTAACCCCAGTACTCAGGTCACAAACAAAATGGGAACAGATACAGTGTATGTCGGCCAGGACTACCCTTCTGGCTT
ATCAAAACGGGTACCAGCACGGTTAGTGGCGGGACCGATGCTGCGAGAGCGAAGCTGTCACGCCCATGTGTTTAGGGCTGGACACATGTGGAACTGGCGAACCA
GCCTTCCGAGCGGGCGCTGGGACCAGCCCGCTTTGGAGAAGTCTCGGGTCCTAACCCGGTCGGTGGCGACGGCCACCGACCCCGAAATTACCTCTTACCCAGGA
AAGTCCGTATCGACAAGTACGCAGGTTCAGGAGGAGGACTGGTGTAGCCGGGAGAGCGGGTGGATCTCGCCAGGACTTGCTCCTGAAGAACCCTCGGTGGTGTC
CGAAATTACAGCCTCCATGGTAGCGACAATGAGGGTAGCAACCGAGGAGGTCGTGCTGGAACCACAGCCTGAACAGGTCGTCACAATACTGCCGGAGCATGGTC
GAAACGTTCCTCCGGGGCTGGCAGAACAGGACACCGCCAGCCCCATAGAAGTCTCGGTGCTCCTCCCAGACCTCGCTGAGAACTGCCCATTGTGTGGCGTGCCG
AGCGGGGGCCTACGCTTGCTCGGGAAGCATTTTGCTGTCCGACATGCGGGGGTGCCTGTAACGTATGAGTGCCGTAAGTGTGCGTGGCGGAGCCCCAACAGCCA
CTCAATCTCGTGTCACGTCCCCAAATGCCGGGGGCGTGCGCGGATGCCCAGTGGCGATCCAGGGATCGCCTGCGATCTCTGTGAAGCCCGGTTTGCCACGGAGG
TTGGGGTCGCCCAACACAAGCGGCACGTTCATCCGGTGGAGTGGAACAAGGTGAGGCTGGAAAGGAGAGGTGCGCGCGGAGGGGGAATTAAGGCGACGAAGCTC
TGGAGTGTAGCGGAGGTAGAGACGCTAATCCGGCTCATCCGTGAGCACGGAGATTCAGGTGCCACTTACCAGCTCATTGCCGATGAGCTGGGAAGGGGCAAGAC
GGCCGAACAGGTGAGGAGTAAAAAGAGGCTCCTGCGCATAGATACGGCAAGCAATAGCCCAGATGATGCAGAGGTTGAGGAGGAGAGGTTGGAATCTCTGGCGG
TTCGGTCCTCGTCACGGTCACCCCCGAGCCTGGTGGCGACCAGGGTCAGGGAGGCAGTTGCCAGGGGTGAATCAGAAGGTGGCGAGGAGATCAGGGCTATTGCT
GCTCTCATTAGGGACGTAGATCAGAATCCTTGTCTGATTGAAACCTCGGCGTCGGACATCATCTCGAAGCTGGGAAGGAGGGTGGATGGGCCCAAGAGACCCAG
GCCCGTTGTCAGAGAACAGACCCAAGAGAAGGGATGGGTAAGGCGGCTTGCCCGGCGGAAAAGGGAGTACAGAGAAGCGCAGTACCTGTACTCAAGGGATCAAG
CAAGGCTGGCGGCCCAGATCCTCGATGGTGCCGCCAGCCAGGAATGCGCCCTCCCGGTGGACCAGGTCTACGGAGCGTTCCGTGAGAAATGGGAAACCGTAGGG
CAGTTCCACGGACTTGGTGAGTTCCGGACGGGTGCACGCGCAGACAACTGGGAGTTCTACTCTCCAATTCTGGCGGCTGAGGTGAAAGAAAACCTAATGAGAAT
GGCTAACGGCACGGCCCCGGGACCAGACAGGATAAGCAAAAAGGCTCTGCTTGACTGGGACCCCCGGGGTGAGCAACTGGCACGGCTGTACACGACGTGGCTGA
TCGGTGGGGTCATACCAAGGGTCTTCAAGGAGTGCAGGACTAAGCTGCTACCGAAATCCAGCGACCCGGTCGAGTTGCAGGACATCGGTGGATGGAGGCCGGTG
ACGATTGGGTCGATGGTGACTAGGCTGTTCAGTCGGATTCTAACGATGAGGCTAACCCGAGCCTGTCCGATCAATCCGAGGCAGCGCGGTTTCTTGGCCTCCTC
GAGTGGATGCGCGGAAAACCTGTTGATCTTTGACGAGATCGTCAGGCGCTCGAGGCGGGACGGGGGGCCGCTGGCAGTGGTGTTTGTGGACTTTGCGAGGGCCT
TTGACTCCATCTCACATGAACATATCCTGTGTGTTCTCGAAGAAGGCGGGCTTGACAGGCACGTTATCGGGTTGATCCGAAACTCGTACGTGGATTGCGTGACC
AGGGTGGGTTGTGTCGAGGGCATGACACCACCAATACAAATGAAGGTTGGAGTGAAGCAGGGAGACCCCATGTCCCCCTTGCTCTTCAACCTGGCTATGGATCC
CCTCATCCATAAACTCGAGACGGCCGGAACTGGACTGAAATGGGGCGATCTTTCAATCGCCACGCTGGCCTTTGCCGACGATCTGGTGCTGGTGAGTGACTCTG
AGGAAGGCATGGGGAGGAGTCTCGGGATTTTGGAGAAGTTTTGCCAACTGACTGGGCTGAGGGTTCAGCCCAGGAAGTGTCACGGTTTCTTTATGGACAAGGGC
GTGGTGAACGGCTGTGGAACCTGGGAAATCTGTGGGTCACCGATCCACATGATTCCCCCGGGGGAATCAGTTCGTTATTTGGGAGTCCAGGTAGGCCCGGGGCG
CGGCGTGATGGAACCGGATCTTATCCCTACGGTCCACACGTGGATCGAAAGGATCTCGGAGGCTCCTCTAAAGCCCTCACAACGCATGAGGGTTTTGAACTCAT
TCGCTCTCCCCCGGATAATTTACCAGGCCGATCTAGGGAAGGTTACGGTAACCAAATTGGCCCAGATAGATGGGATTGTCCGGAAGGCTGTGAAGAAGTGGCTC
CATTTGTCACCATCCACGTGCAATGGACTGCTGTATTCACGGAACCGCGACGGTGGTTTGGGCCTCCTAAAGCTGGAAAGACTAATCCCATCCGTGCGCACGAA
GCGTATCTATCGGATGTCCAGGTCTCCGGATATCTGGACACGGCGAATGACCAGCCATTCTGTGTCAAAATCTGACTGGGAGATGTTGTGGGTCCAAGCGGGAG
GTGAGAGGGGCAGTGCACCTGTAATGGGTGCCGTGGAGGCTGCCCCGACCGATGTGGAGAGATCGCCAGACTACCCAGACTGGCGGCGTGAGGAAAACCTGGCA
TGGTCGGCCCTGCGGGTGCAGGGTGTGGGTGCAGACCAGTTTCGAGGCGACAGGACCAGCAGCTCTTGGATCGCCGAGCCCGCTTCGGTTGGGTTCGCGCAGCG
CCACTGGTTGGCTGCCCTGGCGCTGAGGGCTGGGGTGTATCCGACTCGGGAGTTTCTGGCTCGGGGTAAGGAAAAGTCAGGAGCAGCTTGCAGACGCTGCCCGG
CCAGGTTGGAATCATGTTCACACATACTTGGGCAATGTCCGTTCGTTCAGGCGAACAGAATTGCGAGGCACAACAAGGTGTGTGTGCTCTTGGCCACGGAGGCG
GAGAGGTTCGGCTGGACGGTAATAAGGGAGTTCCGTCTTGAGGACGCCGCTGGCGGTCTCAAGATACCCGACCTGGTTTGCAAGAAGGCCGACACAGTTCTCAT
TGTCGACGTGACCGTCCGGTACGAGATGGATGGAGAGACGCTAAAAAGGGCCGCATCGGAGAAGGTGAAACACTATCTCCCAGTAGGGCAACAGATAACGGACA
AGGTCGGAGGGCGTTGCTTTAAAGTCATGGGGTTCCCTGTAGGTGCTAGGGGAAAGTGGCCGGCGAGCAACAACACAGTTTTGGCTGAGTTAGGCGTCCCTGCA
GGTCGGATGAGGACCTTTGCCAGGCTGGTGAGCCGGAGGACTCTTCTTTATTCTTTGGATATATTGAGGGACTTCATGCGTGAGCCGGCCGGCAGGGGAACTCG
GGTTGCTCTCATCCCTGCGGCAACGGGTGCCGCGAATTGAGGGGGACAGCTGGGAGTCTCGGCATGATTACAAATCTTGCGCTGCACTCGGATGTCGTCCCCGT
GACGGACACATTAATCCGGAAAGCGAGTGGTGACTCGCCTCAAG

Figure S2. Nine clones of R2Ol obtained from medaka fish genomic DNA. **A** Amino acid alignment of nine clones. Different amino acids are shaded in light blue. **B** Schematic diagram of nine R2Ol clones. Arrows indicate amino acid exchanges from all consensus sequences. Green arrows, conserved region exchanges; Black arrow, similar polarity exchanges; Red arrows, other exchanges. **C** Nucleic acid sequences of clone F, used in this study. Green, ORF; Black UTRs. Accession number: LC349444.

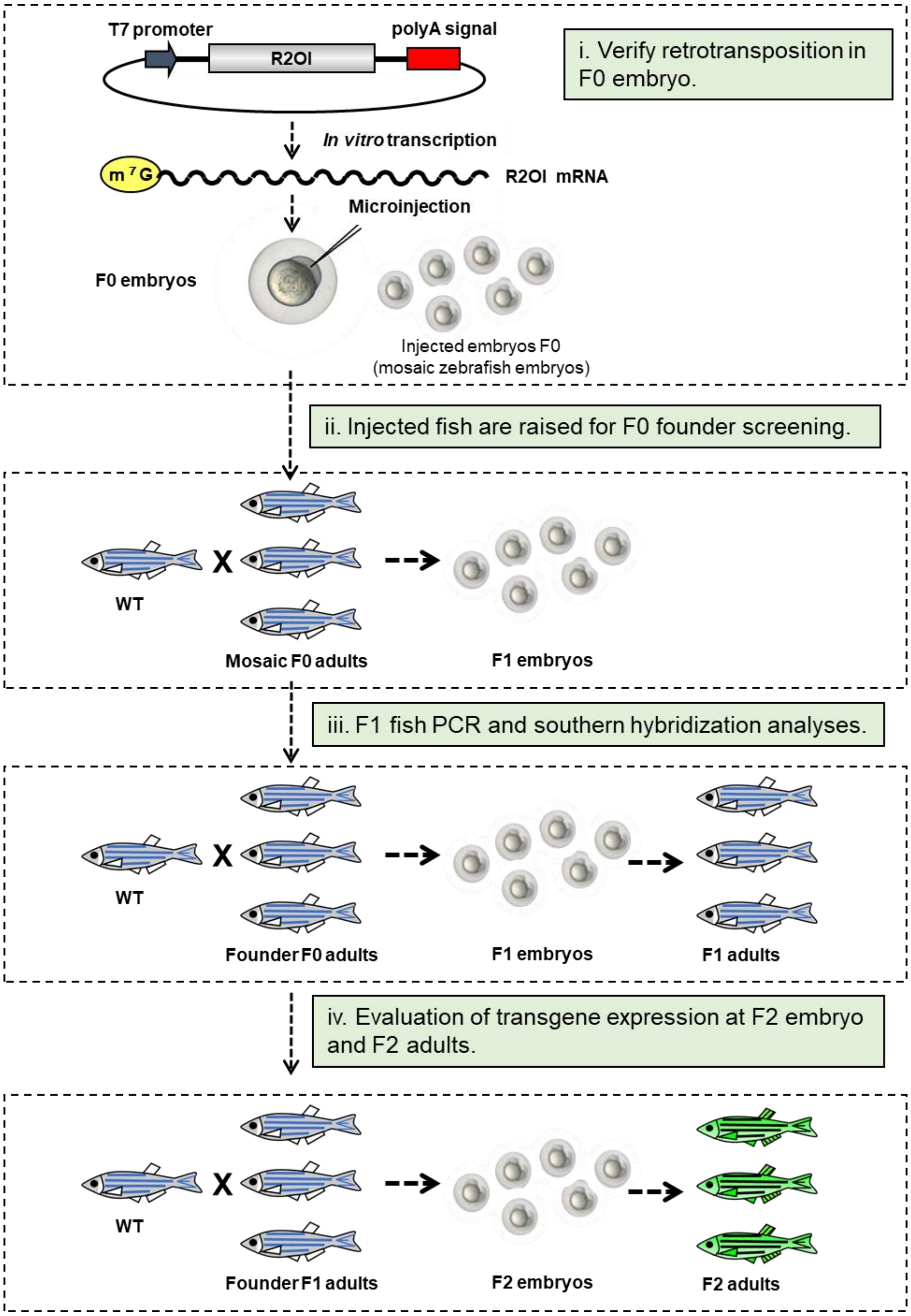

Figure S3. A scheme for transgenesis in zebrafish using R2Ol.

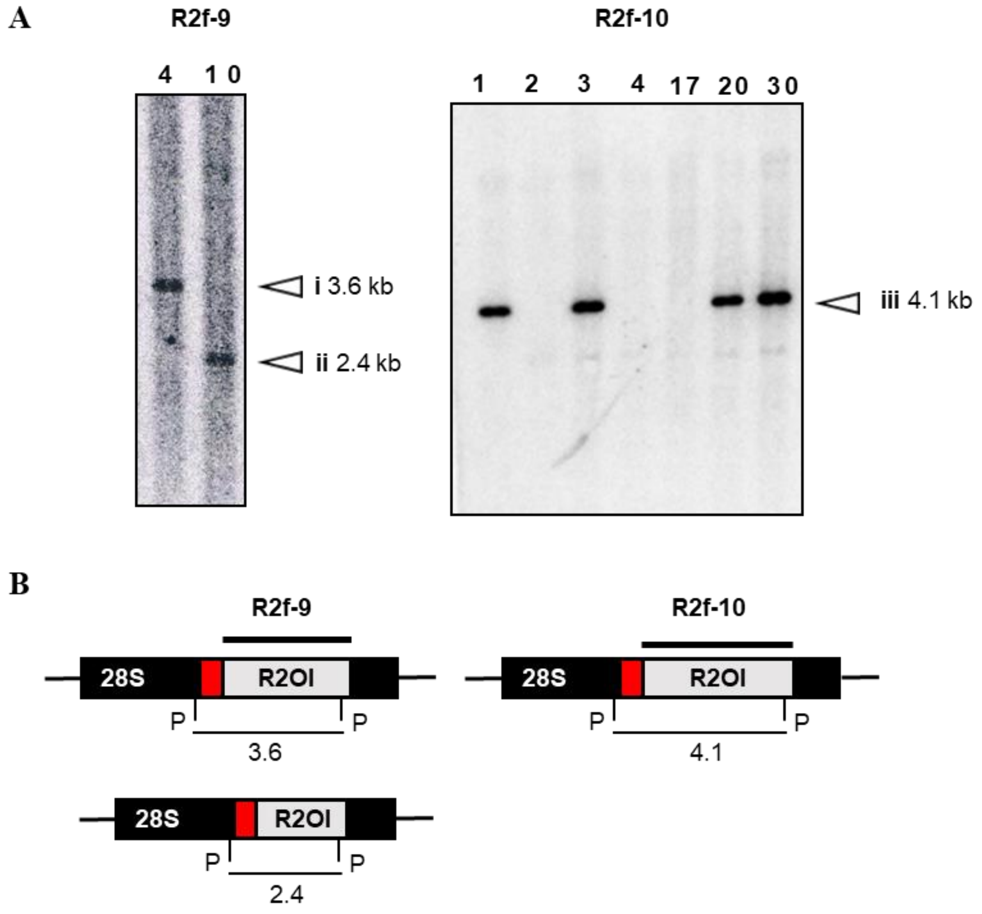

Figure S4. F1 progeny analysis of two founder lines, R2f-9 and R2f-10. **A** F1 fish Southern blot hybridization of representative founders R2f-9 (Left panel) and R2f-10 (Right panel). Genomic DNA were digested with *Pvu*II. **B** Schematic diagram of each R2Ol insertions. P, *Pvu*II site; thick line, probe; thin lines and numbers, signal lengths; Red boxes indicate vector or non-templated sequence.

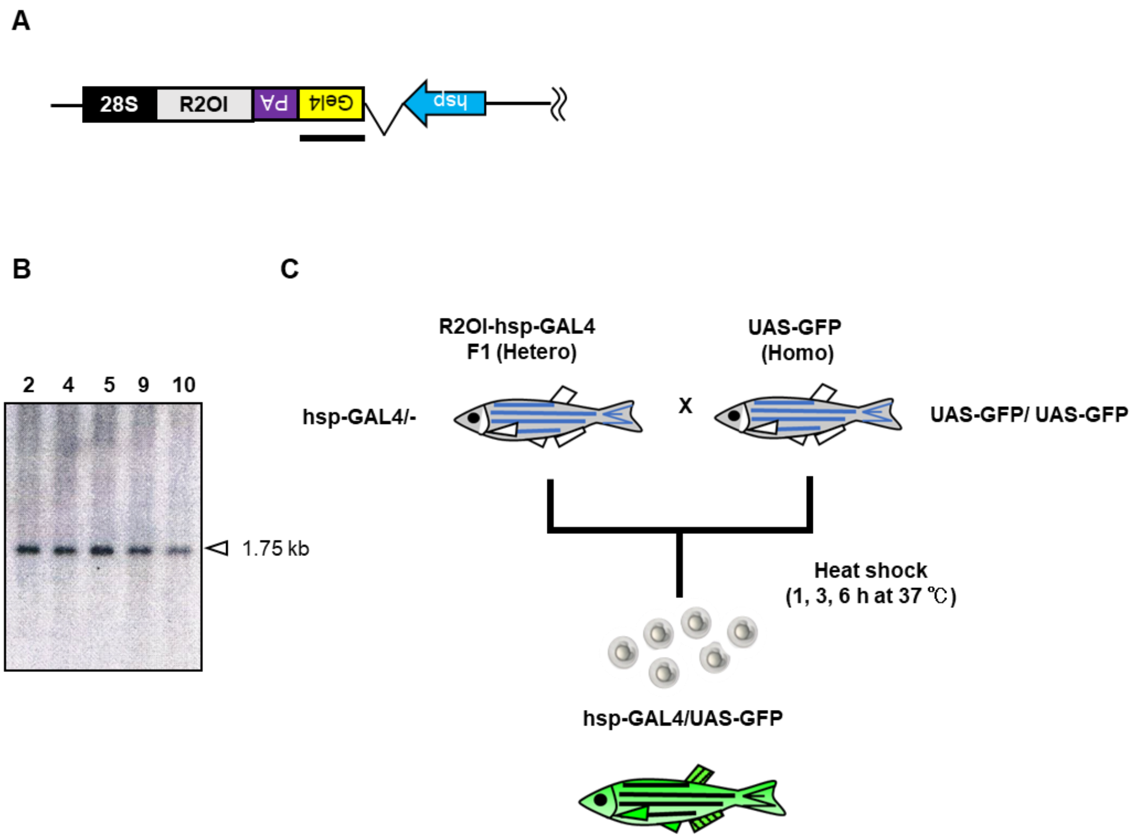

Figure S5. Experimental approach for analysis of hsp-GAL4:UAS-EGFP expression induced by heat shock. **A** Schematic diagram of hsp-GAL4 construct. thick line, probe. **B** Transgenic progeny analysis from R2Ol-hsp-GAL4 founder. **C** Two lines R2Ol-hsp-GAL4 and UAS-GFP were crossed and yielded embryos were heat shocked at 37 ℃ for 1, 3, 6, hours.
