# Supplementary material for: Targeted gene knockin in zebrafish using the 28S rDNA-specific non-LTR-retrotransposon R2Ol
Source: Mob DNA. 2019 May 22;10:23. doi: 10.1186/s13100-019-0167-2 (PMC6530143; doi:10.1186/s13100-019-0167-2)
Supplement: Supplementary file 2 — Table S1. Primer List. (PDF 195 kb) [file 13100_2019_167_MOESM2_ESM.pdf]

Table S1. Primer list

Purpose and primer	Sequence (5′-3′)
**R2Ol cloning**	
R2Ol5′end	TTAAGGCACAGGGGACACAG
R2Ol3′end	AGGCTACTTGAGGCGAGTCA
**Plasmid construction**	
R2Ol-RT(D→V)s	CGCTGGCCTTTGCCGTCGATCTGGTGCTGGTG
R2Ol-RT(D→V)as	CACCAGCACCAGATCGACGGCAAAGGCCAGCG
R2Ol-EN(D→A)s	GGTCTCAAGATACCCGCCCTGGTTTGCAAGAAGGCCG
R2Ol-EN(D→A)as	CGGCCTTCTTGCAAACCAGGGCGGGTATCTTGAGACC
R2Ol (NotI)s	AAAAAGCGGCCGCGGGGACAGCTGGGAGTCTCGG
R2Ol (NotI)as	AAAATGCGGCCGCTCAATTCGCGGCACCCGTT
BglII-EF1p-F	TTTAGATCTAGGGGGATCATCTAATCAAGCAC
BglII-polyA-R	TTTAGATCTGATCTAGAGGATCATAATCAGCCATACC
BglII-hsp-F	TTTAGATCTGAATTCACTGGAGGCTTCCAG
MluI-PA(GFF)	TTTACGCGTGAATTAAAAAACCTCCCACACC
hsp414-R	GTCAGCAACATAAAGTAAATGCCCGTC
hsp154-R	AGTCCTGTCAGGCTGCTCTTTTCTTC
**In vitro transcription**	
T7	TAATACGACTCACTATAGGG
T3	ATTAACCCTCACTAAAGGGCG
R2Ol3′end+r4	GCTACTTGAGGCGAGTCACCACTCG
R2Ol3′end+r10	CATTTGGCTACTTGAGGCGAGTCACCACTCGC
**Retrotransposition assay**	
**3’ junction assay**	
R2Ol s1	AACGGACAAGGTCGGAGGGC
Dr28S a1	GGCCTCCCACTTATTCTACACCC
R2Ol s2	CTCGGGTTGCTCTCATCCCTG
Dr28S a2	CTTCACAGTGCCAGACTAGAGTCAAGC
GFP s1	TGAGCAAAGACCCCAACGAGAAG
GFP s2	ATCACTCTCGGCATGGACGAGC
GFP a1	TGGCACTCCCTAGTGCGTCATAAG
GFP a2	TTACATCTGATAGTGGACCTTAAGCCGAC
**5’ junction assay**	
Dr28S s1	ACCTCCGTCTGGCGTCTTCT
Dr28S s2	TGGCTTAGAACTGGTGCGGACCA
R2Ol a11	GACCATGCTCCGGCAGTATTGTG
R2Ol a12	TTCAGGAGCAAGTCCTGGCGAG
R2Ol a21	GGCCTACCTGGACTCCCAAA
R2Ol a22	AATCGTCACCGGCCTCCATC
R2Ol a31	AAACTGTGTTGTTGCTCGCCGG
R2Ol a32	CCTTCTCCGATGCGGCCCTT
