# Supplementary material for: Targeted gene knockin in zebrafish using the 28S rDNA-specific non-LTR-retrotransposon R2Ol
Source: Mob DNA. 2019 May 22;10:23. doi: 10.1186/s13100-019-0167-2 (PMC6530143; doi:10.1186/s13100-019-0167-2)
Supplement: Supplementary file 3 — Table S2. 5' junction sequence of inserted R2Ol. (PDF 59 kb) [file 13100_2019_167_MOESM3_ESM.pdf]

Table S2 5' junction sequence of inserted R2Ol.

Pattern	28S rDNA	Non-templated	Vector	5'UTR	R2Ol ORF
i	---AACGGCGGGAGTAACTATGACTCTCTTAAGG	TAACTATGACTCTC(14)	CCCCTCGAGGTCGACGGTATC GATAAGCTTGATATCGAATTC GATTGCTCTAGAG(55)	(+1)CGCACAGGAGA--- GTCACAAACAAA(+265)	(+266)ATGGGAACAGAT---GCGAA TTGA(+4093)
ii	---AACGGCGGGAGTAACTATGACTCTCTTCAGG				(+617)TTCAGGTGGTGTACGAAATT ACAGACTCCATGG---
iii	---ACCGGCGGGAGTAACTA(-14)	AACGGTGGAAGGTACCCCCCATGCA CTACTAAACGGTGGACGTACAGCCC ATGCGTAACTAATCAACTGGCCTGA GGAGCAGCCTGCCCACAGCCACCCC TGGTCCGTAGACCAGCGCTCTCGCC GGACCCCAGGGGATAGTCACCCCCC CCGTTTCCCGCTCCCTCGGGAGCGG TACTTGGGCAGGCTCTGTGTCCCCT GTGCGCTCTAGAGCAGTCGAATTCG ATATCAAGCTTATCGATACCGTCGA CCTCGAGGGGGGGGCGGGGTACCCA ATTCG(280)			(+2832)CCCTACGGTCCACACGTGG ATCGAAAGGATCTCGGAGGCTCCTCT AAAGCC---

Red, changed sequence from original sequence. Truncated or deleted sites are indicated by nucleotide numbers.
