# Supplementary material for: Targeted gene knockin in zebrafish using the 28S rDNA-specific non-LTR-retrotransposon R2Ol
Source: Mob DNA. 2019 May 22;10:23. doi: 10.1186/s13100-019-0167-2 (PMC6530143; doi:10.1186/s13100-019-0167-2)
Supplement: Supplementary file 4 — Table S3. Previously reported performance of representative DNA transposons in vertebrate. (PDF 116 kb) [file 13100_2019_167_MOESM4_ESM.pdf]

Table S3. Previously reported performance of representative DNA transposons in vertebrate.

Transposons	Host	Transgenic efficiency	Mosaicism	References
SB	zebrafish	10, 31%	0.4-9, 0.3-16%	(1)
		39, 24, 35%		(2)
		14/58 (24%)		(3)
	mouse	6/42 (14%)		(4)
		7/19 (37%)		(5)
Tol2	zebrafish	1/8 (%)	25/50 (50%)	(6)
		50, 51, 26%		(7)
		16%		(8)
		70, 60, 83%		(9)
	mouse			(10)
PB	mouse	62(34.8%), 1(0.5%), 5(2.7%)/184, 10/96(10.4%)		(11)

References

1. Davidson AE, Balciunas D, Mohn D, Shaffer J, Hermanson S, Sivasubbu S, Cliff MP, Hackett PB, Ekker SC. Efficient gene delivery and gene expression in zebrafish using the Sleeping Beauty transposon. Dev Biol. 2003; 263(2):191–202.
2. Balciunas D, Davidson AE, Sivasubbu S, Hermanson SB, Welle Z, Ekker SC. Enhancer trapping in zebrafish using the Sleeping Beauty transposon. BMC Genomics. 2004;5(1):62.
3. Newman M, Lardelli M. A hyperactive sleeping beauty transposase enhances transgenesis in zebrafish embryos. BMC Res Notes. 2010;3:282.
4. Dupuy AJ, Clark K, Carlson CM, Fritz S, Davidson AE, Markley KM, Finley K, Fletcher CF, Ekker SC, Hackett PB, Horn S, Largaespada DA. Mammalian germ-line transgenesis by transposition. Proc Natl Acad Sci U S A. 2002;99(7):4495–9.
5. Mátés L, Chuah MK, Belay E, Jerchow B, Manoj N, Acosta-Sanchez A, Grzela DP, Schmitt A, Becker K, Matrai J, Ma L, Samara-Kuko E, Gysemans C, Pryputniewicz D, Miskey C, Fletcher B, VandenDriessche T, Ivics Z, Izsvák Z. Molecular evolution of a novel hyperactive Sleeping Beauty

transposase enables robust stable gene transfer in vertebrates. Nat Genet. 2009;41(6):753–61.

6. Kawakami K, Shima A, Kawakami N. Identification of a functional transposase of the Tol2 element, an Ac-like element from the Japanese medaka fish, and its transposition in the zebrafish germ lineage. Proc Natl Acad Sci U S A. 2000;97(21):11403–8.
7. Kawakami K, Takeda H, Kawakami N, Kobayashi M, Matsuda N, Mishina M. A transposon-mediated gene trap approach identifies developmentally regulated genes in zebrafish. Dev Cell. 2004;7(1):133–44.
8. Parinov S, Kondrichin I, Korzh V, Emelyanov A. Tol2 transposon-mediated enhancer trap to identify developmentally regulated zebrafish genes in vivo. Dev Dyn. 2004;231(2):449–59.
9. Kotani T, Nagayoshi S, Urasaki A, Kawakami K. Transposon-mediated gene trapping in zebrafish. Methods. 2006;39(3):199–206.
10. Suster ML, Sumiyama K, Kawakami K. Transposon-mediated BAC transgenesis in zebrafish and mice. BMC Genomics. 2009;10:477.
11. Ding S, Wu X, Li G, Han M, Zhuang Y, Xu T. Efficient transposition of the piggyBac (PB) transposon in mammalian cells and mice. Cell. 2005;122(3):473–83.
